# Lsh regulates LTR retrotransposon repression independently of Dnmt3b function

**DOI:** 10.1186/gb-2013-14-12-r146

**Published:** 2013-12-24

**Authors:** Donncha S Dunican, Hazel A Cruickshanks, Masako Suzuki, Colin A Semple, Tracey Davey, Robert J Arceci, John Greally, Ian R Adams, Richard R Meehan

**Affiliations:** 1 MRC Human Genetics Unit, MRC Institute of Genetics and Molecular Medicine, University of Edinburgh, Edinburgh EH4 2XU, Scotland; 2 Departments of Genetics (Computational Genetics) and Center for Epigenomics, Albert Einstein College of Medicine, 1301 Morris Park Avenue, Bronx, NY, USA; 3 Newcastle Medical School, Framlington Place, Newcastle University, Newcastle Upon Tyne NE2 4HH, England; 4 Room 2 M51 Cancer Research Building, Pediatrics and Oncology, Cellular and Molecular Medicine, Johns Hopkins, Baltimore, MD, USA

## Abstract

**Background:**

DNA methylation contributes to genomic integrity by suppressing repeat-associated transposition. In addition to the canonical DNA methyltransferases, several auxiliary chromatin factors are required to maintain DNA methylation at intergenic and satellite repeats. The interaction between Lsh, a chromatin helicase, and the *de novo* methyltransferase Dnmt3b facilitates deposition of DNA methylation at stem cell genes, which are hypomethylated in *Lsh*^*−/−*^ embryos. We wished to determine if a similar targeting mechanism operates to maintain DNA methylation at repetitive sequences.

**Results:**

We mapped genome-wide DNA methylation patterns in *Lsh*^*−/−*^ and *Dnmt3b*^*−/−*^ somatic cells. DNA methylation is predominantly lost from specific genomic repeats in *Lsh*^*−/−*^ cells: LTR -retrotransposons, LINE-1 repeats and mouse satellites. RNA-seq experiments demonstrate that specific IAP LTRs and satellites, but not LINE-1 elements, are aberrantly transcribed in *Lsh*^*−/−*^ cells. LTR hypomethylation in *Dnmt3b*^*−/−*^ cells is moderate, whereas IAP, LINE-1 and satellite elements are hypomethylated but silent. Repressed LINE-1 elements in *Lsh*^*−/−*^ cells gain H3K4me3, but H3K9me3 levels are unaltered, indicating that DNA hypomethylation alone is not permissive for their transcriptional activation. Mis-expressed IAPs and satellites lose H3K9me3 and gain H3K4me3 in *Lsh*^*−/−*^ cells.

**Conclusions:**

Our study emphasizes that regulation of repetitive elements by Lsh and DNA methylation is selective and context dependent. Silencing of repeats in somatic cells appears not to be critically dependent on Dnmt3b function. We propose a model where Lsh is specifically required at a precise developmental window to target *de novo* methylation to repeat sequences, which is subsequently maintained by Dnmt1 to enforce selective repeat silencing.

## Background

DNA methylation is a crucial epigenetic mechanism associated with stable gene repression, genomic imprinting, X-chromosome inactivation and repetitive DNA silencing [1]. This mark is predominantly found at cytosine in CpG contexts and is also present at lower levels in other cytosine dinucleotide contexts in pluripotent stem cell lineages [2,3]. During early mouse development, genome-wide methylation patterns are established *de novo* by the methyltransferases Dnmt3a and Dnmt3b and are subsequently perpetuated by the maintenance methyltransferase Dnmt1. The importance of DNA methylation is emphasised by the phenotypes of the DNA methyltransferase mouse knockouts: *Dnmt1* knockouts are embryonic lethal between E9.5 and E10.5, and *Dnmt3b* knockouts between E14.5 and E16.5 [4,5]. DNA methylation losses are widespread in the *Dnmt1*^*−/−*^ genome, yet are restricted to minor satellites in the *Dnmt3b*^*−/−*^ genome. By contrast, *Dnmt3a*^*−/−*^ embryos survive to term but are runted and die approximately four weeks after birth - of note, conditional *Dnmt3a*^*−/−*^ knockout germ cells show DNA methylation losses at imprinted loci [5]. The deposition of DNA methylation patterns is regulated by additional factors that include UHRF1 (NP95) and Lsh (also termed Pasg, Hells or Smarca6) [6]. UHRF1 binds to methylated DNA via its SET and RING-associated domain and *Uhrf1*^*−/−*^ mice exhibit embryonic lethality at stage E9.5 [7]. By contrast, *Uhrf1*^*−/−*^ embryonic stem (ES) cells are viable but are hypomethylated at single copy and repeat sequences [7]).

Lsh is a member of the SNF2 subfamily of helicases that are involved in chromatin remodelling and was first cloned in T cell precursors [8]. The Lsh gene has been targeted for deletion in mice by two approaches. The original knockout targeted the ATP-binding site and DExH motif within the helicase domain and exhibited hypomethylation, renal defects, low weight and died shortly after birth. The second Lsh knockout targeted sub-domains II to IV of the helicase domain - these mutants die a number of weeks after birth from a premature aging-phenotype associated with cellular senescence and are also DNA hypomethylated [9,10]. Although *Lsh*^*−/−*^ embryos are viable, DNA methylation losses were observed at single copy genes and repeat sequences [10,11]. Many SNF2 proteins disrupt histone-DNA contacts by ATP-dependent nucleosome mobilisation, which can alter DNA accessibility to transcription factors [12]. Thus, SNF2 activities are critical for the exposure and occlusion of regulatory DNA elements and also determine transcription rates [13]. Consistent with its role as a putative chromatin remodeller, Lsh localises to nuclei and associates with chromatin - however it has weak ATPase activity and is reportedly unable to reposition nucleosomes *in vitro*[14]. DDM1 is the Lsh homologue in *Arabidopsis thaliana*, and like its murine counterpart, its inactivation results in hypomethylated repetitive sequences that exhibit accumulation of the activating associated histone mark H3K4me3 [15]. *In vitro* experiments suggest that DDM1 is an ATPase stimulated by both naked and nucleosomal DNA and promotes chromatin remodelling in an ATP-dependent manner [12]. Recently, it has been demonstrated that DDM1 regulates DNA methylation in plants by mobilising the linker histone H1 to permit methylation deposition [16].

In mammalian DNA, interspersed repeat elements make up nearly half the genome, with long terminal repeat (LTR) endogenous retroviruses (ERVs) comprising 8% to 10% [17]. Without the benefit of suppression mechanisms, active ERVs can transpose to new locations, which is potentially deleterious to regulated gene expression and development [18-20]. LTR-ERV insertions arising from intracisternal A-particle (IAP) retrotransposons account for 10% to 12% of spontaneous mutations in mice [21]. Therefore, the suppression of retrotransposons is vital to normal development and is primarily accomplished via epigenetic silencing mechanisms that include but are not exclusive to DNA methylation [22]. In contrast to dispersed repeats, structural tandemly repeated satellite sequences cluster pericentromerically (major satellites) and overlap the centric constriction (minor satellites) [23]. A common feature in all repetitive DNA element types is the dominant role played by DNA methylation in repeat transcript repression in many biological contexts, which appears to be vital in maintaining genomic integrity [4,21,24]. Strand-specific satellite transcription has been reported to occur subsequent to fertilisation in the early mouse embryo, satellites are mis-expressed in pancreatic tumours and naturally expressed in neuronal lineages [25-27]. Apart from these examples, transcription arising from satellite repeats is heavily restricted in normal development due to the repressive effects of DNA methylation.

Two studies have explored the global effects on DNA methylation in the absence of Lsh in mouse fibroblasts. Tao and colleagues [28] employed methylated DNA immunoprecipitation using 5meC antibodies (MeDIP) combined with a whole-genome tiled array platform covering all autosomes on one biological replicate. A similar approach with reduced genomic coverage was reported that utilised methyl-CpG binding domain purification combined with promoter-specific microarrays [29]. Notably, these studies were not informative of repeat DNA methylation due to the challenges of relating microarray signals from repeat probes with precise genomic locations. Although Lsh contains no recognisable DNA methyltransferase domain, its absence has a marked effect on DNA methylation patterns, therefore the possibility that Lsh is a recruitment protein targeting DNA methylation has been suggested. In this model, Lsh is proposed to recruit the *de novo* methyltransferase Dnmt3b perhaps in concert with polycomb repressor proteins, thus establishing tissue-specific DNA methylation patterns during embryonic development [11,30,31]. This mechanism would predict similarity between the *Lsh*^*−/−*^ and *Dnmt3b*^*−/−*^ molecular phenotypes.

From studies in plants and mice, the major target of Lsh-mediated DNA methylation is the repetitive genome. The complexity of higher eukaryotic genomes makes it reasonable to suspect that DNA methylation must be targeted in an ordered manner and in cooperation with repressive histone modifications. Given the variation of DNA methylation lesions in the knockout models described above, it is currently unclear which epigenetic factors specify DNA methylation targeting at the repetitive complement of the mouse genome. Furthermore, it is unclear if mechanisms of DNA methylation targeting to repeat sequences and single copy genes are similar. Therefore, the goal of this study was to use massively parallel sequencing strategies to understand whether Lsh is influencing repeat methylation through Dnmt3b by comparing profiles in wild-type (WT) and methylation-deficient cells resulting from inactivation of Lsh or Dnmt3b. *HpaII*- tiny fragment enrichment by ligation-mediated PCR coupled to massively parallel DNA sequencing (HELP-seq) is a technique that yields a quantitative readout of DNA methylation at the majority of CpG dinucleotides in *HpaII* restriction sites. We reasoned that HELP-seq analyses of DNA methylation mutants would clarify the specificity of DNA methylation targeting over more than two million CpG sites throughout the mouse genome. Here, consistent with the Dnmt3b-Lsh recruitment model operating at single copy genes, we show that DNA methylation defects in these mutants are strikingly similar at specific repeat classes - importantly, however, the degree of hypomethylation in LTRs is more severe in *Lsh*^*−/−*^ cells than *Dnmt3b*^*−/−*^ cells. Repeat hypomethylation in the absence of Lsh is accompanied by transcription of LTR-ERVs (in particular IAP) and Satellite sequences, with long interspersed nuclear element-1 (LINE-1) repeats being the exception; LINE-1 repeats retain the repressive heterochromatin-associated histone mark H3K9me3. Unlike *Lsh*^*−/−*^ mutants, *Dnmt3b*^*−/−*^ DNA is hypomethylated at LINE-1 and satellites without exhibiting significant repeat expression or loss of repressive chromatin modification at these loci. Crucially, repeat mis-expression in the absence of Lsh also results in alterations in chromatin state at specific repeat classes (LTRs and satellites). We highlight that aberrant IAP transcription leads to the accumulation of IAP protein and the presence of viral-like particles (VLP) in the cytoplasm of Lsh mutant cells - a feature undetected in Dnmt3b mutants. Taken together, Lsh plays an essential Dnmt3b-independent role to ensure that the host genome integrity is protected from potentially deleterious LTR retroelements.

## Results

### HELP-seq reveals discrete repeat compartment hypomethylation in *Lsh*^*−/−*^ and *Dnmt3b*^*−/−*^ mutant fibroblasts

Staining of *Lsh*^*−/−*^ nuclei with a 5-methylcytosine antibody showed a distinct lack of hypercondensed foci (compared to nuclei with WT Lsh protein levels), indicating a dramatic loss of DNA methylation at DNA loci enriched for heterochromatic repetitive element loci (Figure S1a in Additional file 1). To understand the impact of *Lsh*^*−/−*^ loss on repeat DNA methylation we used HELP-seq [32], which we have previously used to assay the methylation profile of WT mouse embryonic fibroblasts (MEFs) [33]. HELP-seq produces a quantitative methylation score at a large proportion of the approximately 2.1 million CCGG sites (*HpaII* site) in the mouse genome [32]. A representation of unmethylated *HpaII* sites is created and an *MspI* (insensitive to methylation at CCGG) library is prepared in parallel as a control for experimental variability and copy number heterogeneity. HELP-seq has advantages over other methylation profiling methods: MeDIP is biased to regions of low CpG density, and isolation of methylated DNA by affinity capture with recombinant methylated DNA binding proteins (that is MBD2) favours regions of higher CpG density and relies on consistent batch production of active recombinant protein [34]. Moreover, HELP-seq is able to sample DNA methylation at CCGG sites ‘genome-wide’ and is therefore compatible with assaying both intergenic and repeat DNA methylation.

**Figure 1:**
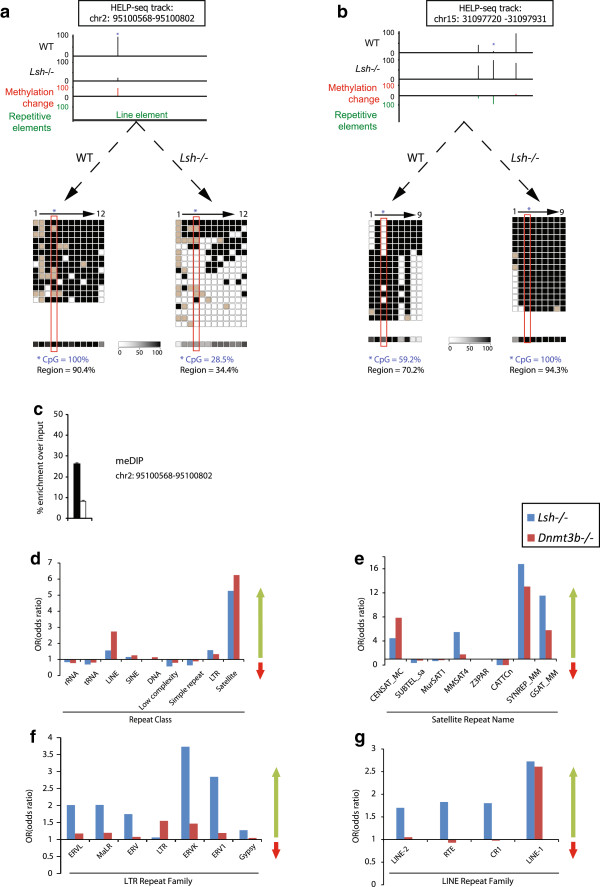
**HELP**-**seq of repetitive genome in mouse. (a)** An example of HELP-seq validation. Top: a HELP-seq browser track showing mouse chr2:95100568–951000802 (genomic build mm9) and loss of methylation at the indicated (asterisk) LINE-1. Bottom: bisulfite sequencing validating the HELP-seq profile for the same LINE-1. Average methylation levels are indicated by methylation heat map. **(b)** Second example of HELP-seq validation. Top: a HELP-seq browser track showing mouse chr15:31097720–31097931 (genomic build mm9) and gain of methylation at indicated (asterisk) genomic locus. Bottom: bisulfite sequencing validating the HELP-seq profile for the same locus. Bisulfite percentages calculated with bisulfite sequencing DNA methylation analysis. For (a) and (b), black square = methylated; white square = unmethylated; grey square = unknown. **(c)** Methylated-DNA immunoprecipitation of the LINE-1 region at chr2:95100568–951000802. Black = WT; white = *Lsh*^*−/−*^ mutant. **(d)** Analysis of genome-wide HELP-seq DNA methylation differences at repeat classes between cell line pairs: WT and *Lsh*^*−/−*^ cells, and *Dnmt3b*^*+/−*^ and *Dnmt3b*^*−/−*^ cells. Scale is the calculated odds-ratio (Figure S2 in Additional file 1). Odds ratio >1 hypomethylation in *Lsh*^*−/−*^ or *Dnmt3b*^*−/−*^ indicated by green arrow; odds ratio <1 hypermethylation in *Lsh*^*−/−*^ or *Dnmt3b*^*−/−*^ indicated by red arrow*.***(e)** Analysis of genome-wide HELP-seq DNA methylation differences at repeat class satellites parsed by satellite repeat name. **(f)** Analysis of genome-wide HELP-seq DNA methylation differences at repeat class LTRs parsed by LTR repeat name. **(g)** Analysis of genome-wide HELP-seq DNA methylation differences at repeat class LINEs parsed by LINE repeat name. Blue = *Lsh*^*−/−*^ and WT comparison; red = *Dnmt3b*^*−/−*^ and *Dnmt3b*^*+/−*^ comparison.

**Figure 2:**
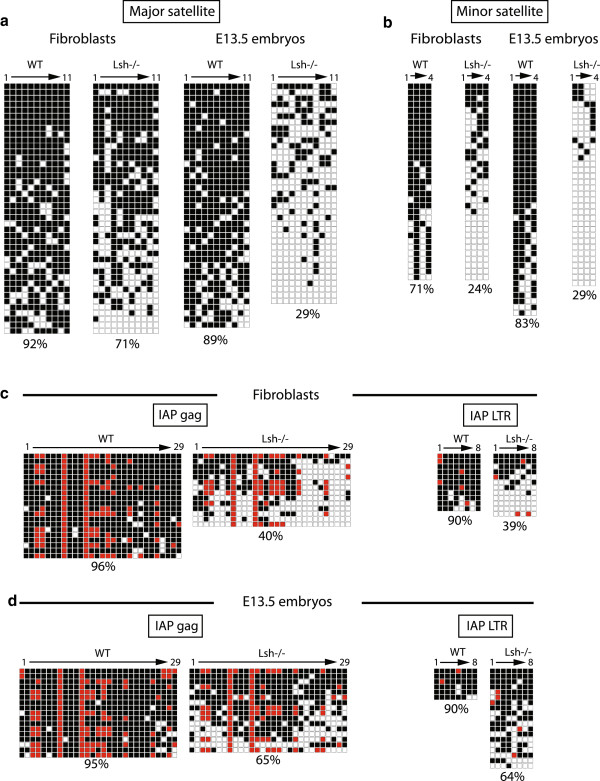
**Bisulfite sequencing of mouse satellite DNA and intracisternal A**-**particle long terminal repeat. (a)** Bisulfite sequencing pattern at the mouse pericentromeric major satellite. **(b)** Bisulfite sequencing pattern at the mouse centromeric minor satellite. **(c)** Bisulfite sequencing pattern at mouse IAP *gag* gene. **(d)** Bisulfite sequencing pattern at mouse IAP LTR. Percentage methylation and cell types indicated. Black square = methylated; white square = unmethylated; red square = non-consensus CpG. See Figure S6 in Additional file 1 for bisulfite primer locations.

Using established HELP-seq analysis methodologies [35] we generated a panel of hypomethylated *HpaII* CCGG sites in *Lsh*^*−/−*^ cells, one example of which is highlighted in Figure 1a. This *HpaII* site was located (chr2:95100643–95100646) in a repeatmasker annotated LINE-1 and our HELP-seq analysis showed dramatic loss of methylation (also observed in *Dnmt3b*^*−/−*^ DNA, Figure S2a, b in Additional file 1). To validate the HELP-seq, we performed bisulfite sequencing flanking this *HpaII* site, which revealed a loss of methylation at this precise site (from 100% to 28.5%). Of particular interest was the observation that non-*HpaII* CpG dinucleotides flanking this HELP-seq tagged *HpaII* site also showed a trend of hypomethylation (from 90.4% to 34.4%). In addition to hypomethylated sites, our analysis revealed HELP tags that acquired methylation in *Lsh*^*−/−*^ genomic DNA relative to WT levels, in agreement with previous results that used affinity purification of methylated DNA in conjunction with promoter microarrays [29]. Bisulfite sequencing of these sites, which did not generally overlap repeatmasker annotations, underlined the validity of HELP-seq (Figure 1b). In addition, the independent technique of MeDIP at a hypomethylated site and bisulfite sequencing of various repeats also validated HELP-seq hypomethylation (Figure 1c and Figure 2). Taken together, these results validate the HELP-seq approach in *Lsh*^*−/−*^ and *Dnmt3b*^*−/−*^ cells and imply that this strategy has the power to identify hypomethylated CpG clusters in these mutants.

Because of the inherent challenges in mapping repetitive DNA sequences, we devised the strategy detailed in Figure S2c in Additional file 1. Briefly, raw *HpaII* HELP library reads were processed and mapped to the mouse genome (build mm9) using Bowtie [36] followed by read parsing on the basis of their overlap (reads with no overlap were not analysed further) to the murine repeatmasker annotation. Using custom Perl scripts, reads were counted in respective repeat classes, 2 × 2 contingency table odds ratios were computed followed by rigorous statistical testing (see Materials and methods section). We observed increased odds ratio scores (indicative of hypomethylation in *Lsh*^*−/−*^ cells) in specific repeat classes: LINE, LTR and satellite (Figure 1d). To test the robustness of HELP-seq and our repeat pipeline, we performed three replicates of HELP-seq libraries from WT and *Lsh*^*−/−*^ DNA isolations, which showed highly reproducible methylation results (Figure S2d, e in Additional file 1). Interestingly, we observed a similar result in the *Dnmt3b*^*+/−*^ | *Dnmt3b*^*−/−*^ library comparison, which is consistent with previous investigations implicating Lsh as a recruitment protein for *de novo* methylation (Figure 1d) [11]. We performed a more specific parsing of our HELP-seq data by subdividing the repeat classes LTR and LINE into their respective repeat families and SAT (satellite) repeat sub-classes. This analysis revealed similar patterns of hypomethylation at satellite repeats in *Lsh*^*−/−*^ and *Dnmt3b*^*−/−*^ DNA (Figure 1e). The degree of hypomethylation varied within repeat classes: for example, some classes (CENSAT_MC) were more hypomethylated in *Dnmt3b* mutant cells compared to *Lsh*^*−/−*^ cells while others were less hypomethylated. Furthermore, we noticed that hypomethylation at LTR repeat families was more severe in *Lsh*^*−/−*^ compared to *Dnmt3b*^*−/−*^ (Figure 1f). Lastly, hypomethylation was detected at all LINE families in *Lsh*^*−/−*^ DNA; however, hypomethylation was restricted to the LINE-1 repeat sub-family in *Dnmt3b*^*−/−*^ DNA (Figure 1g). We noted that some repeat classes yielded odds ratio scores <1 indicating hypermethylation consistent with our initial HELP-seq analysis (Figure 1b) and the redistribution of DNA methylation reported in other Lsh studies [28,29]. Taken together, our HELP-seq data show that hypomethylation is greater at LTRs and LINEs in the absence of Lsh than Dnmt3b, and identify the hypomethylation overlaps and differences that exist between these mutant backgrounds.

A previous report describing expression changes in *Lsh*^*−/−*^ mutant embryonic tissues using custom cDNA microarrays indicated a possible link between Lsh and LTR repeat silencing [37]. Here, our HELP-seq approach has extended this analysis by linking Lsh-mediated DNA methylation patterns to uniquely mapped repeat sequences on a genome-wide scale. We have shown that Lsh regulates DNA methylation signatures at specific repeat classes (LTRs, LINEs and satellites) while not appearing to play a major role in methylation pattern regulation of other repeat classes. In line with the cooperation observed between Lsh and Dnmt3b at some single copy gene promoters, we found that Lsh and Dnmt3b both regulated satellite DNA methylation. However Lsh played a greater role in the regulation of LTR and LINEs, suggesting Dnmt3b-independent roles for Lsh in repeat methylation.

### Bisulfite sequencing reveals repeat sequence hypomethylation in *Lsh*^*−/−*^ fibroblasts and embryos

Although Lsh is a regulator of DNA methylation, it is dispensable for mouse embryonic development. This is in contrast to *Dnmt1*^*−/−*^ embryos (death by E9.5 to E10.5), *Dnmt3b*^*−/−*^ embryos (death between E14.5 and E16.5) and *Uhrf1*^*−/−*^ embryos (severe phenotype at E9.5) [4,7,38]. Lsh has been targeted for deletion by two independent strategies focusing on the helicase domain, but importantly the methylation defects in these mutants are very similar [9,10]. Inspection of expression microarray data and immunoblotting on *Lsh*^*−/−*^ extracts showed that the methylation defects were not caused by loss of expression of Dnmt1, Dnmt3a or Dnmt3b protein or RNA expression levels (Figure S1c in Additional file 1 and further data not shown). One explanation for the late phenotypic effects of Lsh deletion may be related to later embryonic hypomethylation events than those observed in *Dnmt1*^*−/−*^, *Dnmt3b*^*−/−*^ or *Uhrf1*^*−/−*^ embryos. To test this, we performed bisulfite sequencing on the repeat classes that exhibited the most severe hypomethylation by HELP-seq in adult fibroblasts and embryos isolated at the mid-developmental stage E13.5. Percentage methylation values for loci analysed were calculated using bisulfite sequencing DNA methylation analysis (see Materials and methods). First, we examined methylation at the tandemly repeated arrays of satellite DNA overlapping and adjacent to the centromere. In fibroblasts derived from adult mouse tail-tips, loss of methylation in the absence of Lsh was substantial (92% to 71%) for the pericentromeric major satellite (Figure 2a) and was severe in DNA isolated from E13.5 whole embryos (89% to 29%). Analysis of the centromeric minor satellite repeat indicated severe hypomethylation in *Lsh*^*−/−*^ embryonic tissues and adult *Lsh*^*−/−*^ genomic DNA (Figure 2b). Our bisulfite analyses show that *Lsh*^*−/−*^ associated hypomethylation is not limited to cultured cells, occurring as early as the E13.5 embryonic stage, and imply that appropriate methylation of satellite repeats is not required for embryo survival.

It has been previously observed that IAP retroviral elements are regulated via methylation by Dnmt1 and Dnmt3b [39,40], therefore we focused on the methylation status of this subtype of LTR retrotransposon. Initially, we sequenced across the IAP *gag* gene due to its CG-rich nature. It showed high levels of methylation in WT DNA that was reduced to 40% in *Lsh*^*−/−*^ fibroblasts and 65% in E13.5 embryos (Figure 2c, d, left panels). Because of the regulatory functions on IAP activity of the LTR [41], we carried out a similar analysis on the LTR regions that flanked the 5′ and 3′ ends of the IAP retroviral cassette. We found that methylation of IAP flanking LTRs mirrored the IAP *gag* gene in each genomic DNA analysed, that is, methylation was reduced in *Lsh*^*−/−*^ fibroblast and E13.5 genomic DNA (Figure 2c, d, right panels). Notably, in *Dnmt3b*^*−/−*^ DNA, consistent with our HELP-seq analysis, IAP LTR and IAP *gag* sequences were only partially hypomethylated (LTR: 90% down to 73%; *gag*: 94% down to 88%) relative to the effect observed in *Lsh*^*−/−*^ genomic DNA (Figure S2f in Additional file 1). These data further validate our HELP-seq methylation screen and suggest that Lsh and Dnmt3b have differing influences on repeat methylation. In addition, our results underline that DNA methylation defects precede the lethality of Lsh mutant new born mice previously reported for this mutation [9,10].

### RNA-seq analysis of DNA methylation mutants reveals specificity in intracisternal A-particle element transcriptional control

DNA methylation is widely believed to repress transcription of potentially detrimental elements that have the capacity to impact genome stability, including tandem repeat satellites, LTRs (that is, IAP) and non-LTR (that is, LINE-1) dispersed repeats [41]. A study using customised cDNA microarray technology reported that *Lsh*^*−/−*^ embryonic tissues mis-express cDNA clones, some of which contain repeatmasker annotated sequences [37], although this study did not unequivocally link transcription of these repeat-containing cDNAs to hypomethylation events at the same loci in the murine genome. To directly link the HELP-seq DNA methylation defects at repeats to transcription, we employed RNA-seq on WT and *Lsh*^*−/−*^ fibroblasts. To measure relative repeat sequence transcript abundance, we limited our pipeline to unambiguously mapped unique hits from the TopHat [42] RNA-seq mapping package. We chose the unique hit parameter as this permitted direct association of transcription and DNA methylation (HELP-seq) at single copy genomic locations. Processed aligned reads were parsed against the repeatmasker annotation in Galaxy [43] and differential analyses were performed in the R programming environment [44] using the EdgeR package [45]. Given the IAP and LINE-1 HELP-seq methylation results, we focused on these elements for differential analysis. Notably, a similar analysis for satellites was hampered by centromeric and pericentromeric sequences being under-represented in the sequenced mouse reference genome (mm9); however, confirmatory quantitative PCR (qPCR) satellite analysis indicated robust activation of satellite transcription (see below).

**Figure 3:**
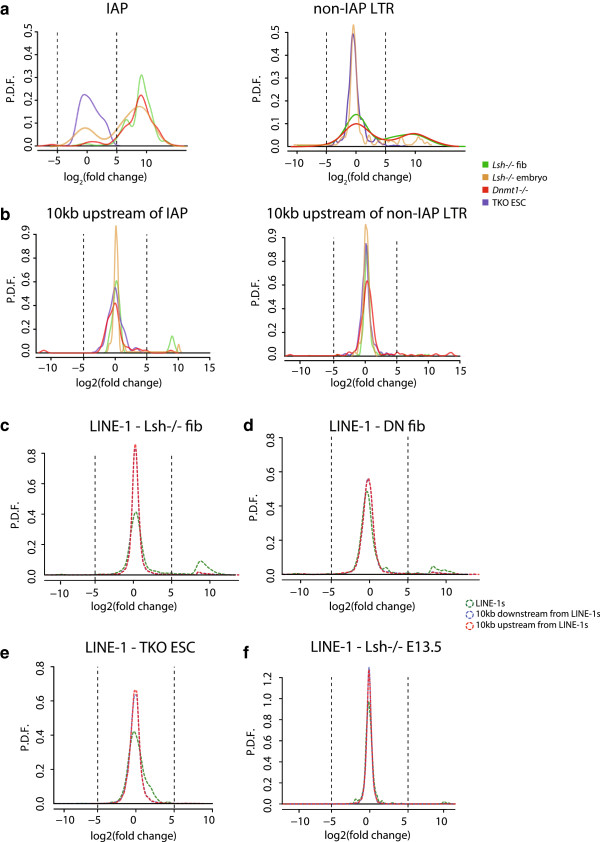
**Differential RNA**-**seq analysis of mouse intracisternal A**-**particle and long interspersed element**-**1 in DNA methylation mutants. (a)** Left: IAP fold-change (x-axis) plotted against probability density function (y-axis) in indicated cell types. Right: non-IAP LTR fold-change plotted against probability density function in indicated cell types. **(b)** Left: 10 kb upstream IAP fold-change plotted against probability density function in indicated cell types. Right: 10 kb upstream non-IAP fold-change plotted against probability density function in indicated cell types. **(c)** LINE-1 fold-change plotted against probability density function in *Lsh*^*−/−*^ fibroblasts. **(d)** LINE-1 fold-change plotted against probability density function in *Dnmt1*^*−/−*^ fibroblasts. **(e)** LINE-1 fold-change plotted against probability density function in TKO ES cells. **(f)** LINE-1 fold-change plotted against probability density function in E13.5 embryos. Vertical lines indicate −5 and +5 log_2_-fold changes. X-axis is log_2_-fold change in repeat RNA expression; y-axis is probability density function (P.D.F.). fib, fibroblasts; TKO ESC, triple knockout embryonic stem cells.

Initially, we validated our RNA-seq pipeline using two published RNA-seq datasets. Consistent with these previous studies, our RNA-seq analysis showed aberrant expression of LTRs in *Dnmt1*^*−/−*^ MEFs, in contrast to Dnmt triple knockout (TKO) stem cells, which show little LTR mis-expression (Figure 3a) [33,46]. Importantly, differential RNA-seq analyses for IAP and non-IAP LTRs in *Lsh*^*−/−*^ cells showed a marked skew towards upregulation of IAPs. Interestingly, although a subset of non-IAP LTRs showed a moderate skew towards activation (Figure 3b) in *Lsh*^*−/−*^ cells, this effect was not as pronounced as IAP elements for which the majority of informative loci showed de-repression (Figure 3a). Additionally, we analysed RNA-seq from WT and *Lsh*^*−/−*^ E13.5 whole embryos and noted that the majority (>60%) of IAP loci were skewed towards activation in mutant embryos (Figure 3a), indicating that hypomethylation and aberrant transcription occurred at this stage of embryonic development. Our data suggest that LTR sequences are activated in the absence of Lsh and that the IAP subtype is particularly sensitive in this mutant background.

Examples of read-through transcription initiating from the 5′ of IAPs and terminating in unique sequences 3′ to the end of the IAP have been reported in the livers of aging mice [47]. To address whether transcriptional activity at LTRs is due to read-through transcription initiating from non-LTR regulatory elements or from *bona fide* LTR transcripts, we modified our RNA-seq pipeline to examine genomic intervals of the equivalent width either 10 kb upstream or 10 kb downstream from LTR annotations in the reference mouse genome. In contrast to the abundance of RNA-seq reads directly overlapping IAP annotations (Figure 3a), the majority of RNA-seq reads either upstream or downstream (Figure S3a in Additional file 1) of IAPs showed little change in transcription between cell type pairs (Figure 3b). In a number of cases, the minor group of IAPs showing upregulation remote from IAP annotated sequences could be explained by the 10 kb shifted coordinates overlapping a neighbouring repeat - frequently a neighbouring IAP LTR (Figure 3b and Figure S3a in Additional file 1). From this we can conclude that loss of DNA methylation in *Lsh*^*−/−*^ cells leads to LTR transcription that is restricted to these loci with little influence on neighbouring genes.

Given the parallel between IAP hypomethylation and transcription, we performed a similar series of analyses using LINE-1 repeatmasker annotated loci in our RNA-seq pipeline. Surprisingly, despite the hypomethylation of LINE-1 by HELP-seq (Figure 1g), only a minor fraction of informative LINE-1 repeats were activated in the absence of Lsh (Figure 3c). A lack of LINE-1 activation was mirrored in Dnmt1-deficient fibroblasts and was completely absent from TKO stem cells (Figure 3d, e). Notably, LINE-1 transcription was not detected in *Lsh*^*−/−*^ E13.5 embryos, ruling out the possibility that the lack of LINE-1 transcription in fibroblasts was due to culture conditions (Figure 3f). Similar to Dnmt1-mutant MEFs and TKO stem cells, these analyses suggest that hypomethylation of LINE-1s due to loss of Lsh is insufficient for their de-repression.

**Figure 4:**
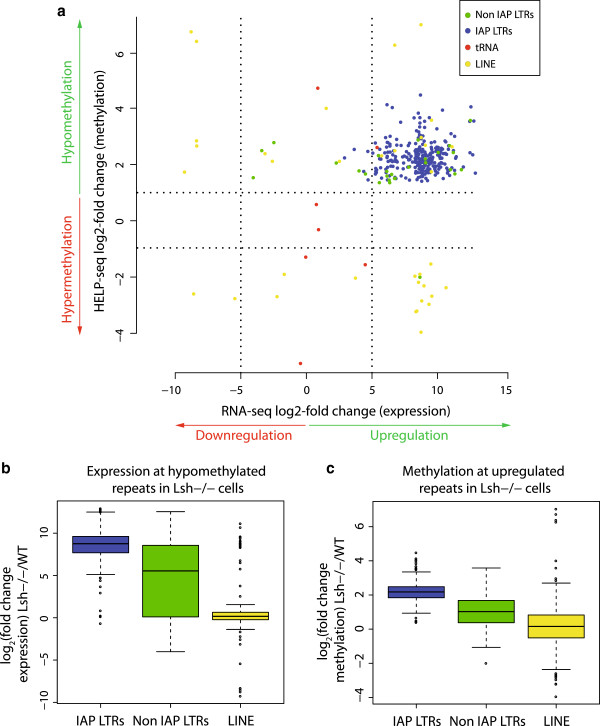
**DNA hypomethylation and expression of long terminal elements correlate in *Lsh***^***−/− ***^**mutants. (a)** Plot of RNA-seq expression changes versus HELP-seq DNA methylation changes in *Lsh*^*−/−*^ versus WT cells. Repeat classes plotted are indicated in various colours (see key). Significant differences detected using EdgeR in RNA-seq reads sequenced between *Lsh*^*−/−*^ and WT cells are plotted against significant changes (Fisher’s t-test and Benjamini-Hochberg multiple testing) in HELP-seq reads. Vertical dashed lines indicate −5 and +5 RNA-seq log_2_-fold change thresholds and horizontal dashed lines indicates −1 and +1 log_2_-fold change HELP-seq threshold. **(b)** Boxplot of RNA-seq log_2_-fold changes at hypomethylated repeat classes. HELP-seq data was filtered for significantly hypomethylated unique repeats and the distribution of their RNA-seq expression levels are shown in boxplot form. **(c)** Boxplot of HELP-seq log_2_-fold changes at upregulated unique repeats. RNA-seq data was filtered for significantly upregulated unique repeats and the distribution of their HELP-seq methylation levels are shown in boxplot form.

We directly compared methylation and transcription changes at unique repeat sequences in the absence of Lsh by plotting transformed HELP-seq fold changes against transformed RNA-seq fold changes (Figure 4a). This demonstrated that virtually all upregulated IAP LTRs showed hypomethylation in *Lsh*^*−/−*^ HELP-seq libraries (compared to WT)*.* Non-IAP LTRs showed a similar upregulation and hypomethylation; however, unique tRNA and LINE sequences showed no strong association (Figure 4a). Examination of all LINE-1 unique sequences (including statistically non-significant data) revealed that most LINE-1s did not change in expression (Figure 3c) and that there was no association between hypomethylation and upregulation at these elements (Figure S4 in Additional file 1).

To further associate methylation state with transcriptional activity, we selected significantly hypomethylated unique repeat sequences (*P* <0.05) and examined the distribution of associated expression changes. As shown in Figure 4b, IAP LTR and non-IAP LTR showed both a higher expression change distribution and median than LINE sequences. The reciprocal comparison (selection of unique significantly upregulated repeat sequences; *P* <0.05) indicated that hypomethylation was more pronounced at IAP and non-IAP LTR (greater hypomethylation distribution and median) than unique LINE sequences (Figure 4c). Comparison of upregulated unique LTR sequences showed that a greater overlap existed between non-IAP LTRs in *Lsh*^*−/−*^ and *Dnmt1*^*−/−*^ cells than IAP LTRs, suggesting a higher degree of specificity for IAP LTRs regulation (Figure S3c in Additional file 1). Indeed, differentially expressed *Lsh*^*−/−*^ IAPs were generally further from neighbouring genes than *Dnmt1*^*−/−*^ IAPs, whereas differentially expressed non-IAP LTRs were closer to neighbouring genes than the genome average (Figure S3d in Additional file 1). Interestingly, comparison of *Lsh*^*−/−*^ and *Dnmt1*^*−/−*^ IAPs with a published dataset that classified all mouse LTRs into clades indicated no preferential activation of particular sub-clades (Figure S3e in Additional file 1). Taken together, our analysis suggests a direct link between hypomethylation and aberrant transcription in *Lsh*^*−/−*^ mutant fibroblasts at unique IAPs and non-IAP LTRs but not at LINE sequences.

**Figure 5:**
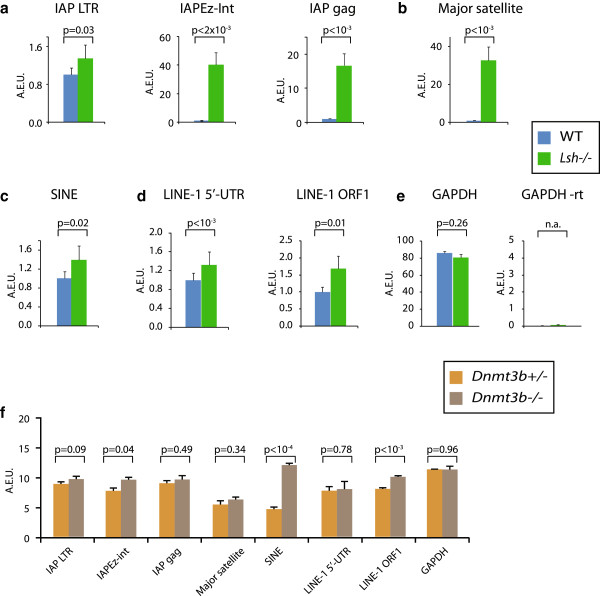
**Validation of repeat RNA**-**seq data by quantitative RT**-**PCR. (a)** qRT-PCR expression of IAP sequences in WT and *Lsh*^*−/−*^ cell showing major transcriptional de-repression at IAP-*gag* and IAPEz-int. **(b)** Similar to (a), marked de-repression of mouse major satellite transcripts. **(c)** Short interspersed element sequences show low expression in all cell types (note scale). **(d)** LINE-1 5’UTR and ORF1 transcripts show little de-repression in mutant cell types. **(e)** GAPDH expression loading control for cell types analysed showing appropriate levels of this constitutive transcript. -rt shows undetectable signal in the absence of reverse transcriptase. **(f)** Indicated repeat expression levels in *Dnmt3b*^*+/−*^ and *Dnmt3b*^*−/−*^ MEFs. Blue = WT fibroblasts; green = *Lsh*^*−/−*^ fibroblasts; orange = *Dnmt3b*^*+/−*^ MEFs; brown = *Dnmt3b*^*−/−*^ MEFs. Experiments represent triplicate analysis. See Figure S6 in Additional file 1 for qRT-PCR primer locations. Expression level units are arbitrary and are all normalised to GAPDH expression levels. A.E.U, arbitrary expression units, SINE, short interspersed nuclear element; UTR, untranslated region.

We validated our RNA-seq analysis using candidate repeat expression by qRT-PCR, which was normalised to the expression of the house-keeping gene *Gapdh* (Figure 5e); reactions without reverse transcriptase were negative by qRT-PCR. Given the dramatic effects at IAP LTRs by HELP-seq and RNA-seq, we initially examined RNA expression within LTRs flanking IAPs and the IAP *gag* gene. Mis-expression was limited to the IAP *gag* gene with no de-repression observed in the regulatory LTR flanking the IAP open reading frames (ORFs) (Figure 5a). The trend of IAP *gag* activation was also seen in *Lsh*^*−/−*^ E13.5 embryos; indicating that IAP deregulation occurred during development and was not limited to differentiated adult fibroblasts (Figure S5a in Additional file 1). In general, the magnitude of IAP expression was lower in embryos compared to fibroblasts, possibly due to the cellular complexity of E13.5 tissue in comparison with the homogenous nature of cultured fibroblasts. *In vivo* major satellite expression is largely restricted to a brief burst during the initial cleavages of the early mouse zygote [26]. HELP-seq and bisulfite sequencing suggested that *Lsh*^*−/−*^ cells may be permissive to satellite transcription. This was confirmed by qRT-PCR, which showed considerable major satellite expression in *Lsh*^*−/−*^ fibroblasts and embryos and the absence of expression in a WT background (Figure 5b and Figure S5b in Additional file 1). We detected no bias in strand preference for mis-expression of satellite transcripts in *Lsh*^*−/−*^ cells (Figure S5f in Additional file 1). Our study demonstrates that unique IAP LTRs lose methylation and become transcriptionally active.

In agreement with our HELP-seq data that suggested modest hypomethylation at short nuclear interspersed elements (SINEs), we observed little activation of these transcripts (Figure 5c). Although LINE-1 sequences were hypomethylated by HELP-seq, they were weakly de-repressed when assayed by qRT-PCR over the LINE-1 5′-untranslated region (UTR) and ORF1 regions (Figure 5d) and RNA-seq (Figure 3c-f), suggesting additional repressive mechanisms at LINE-1s. Our qPCR data validated the RNA-seq global approach and also indicates discrete specificity for the repetitive DNA compartments controlled by Lsh. Because the Lsh and Dnmt3b HELP-seq overlapped in part, we carried out similar qRT-PCR analyses on *Dnmt3b*^*−/−*^ MEFs compared to *Lsh*^*−/−*^ cells; IAP elements and major satellite transcripts were not strongly mis-expressed in *Dnmt3b*^*−/−*^*cells* (Figure 5f). Mis-expression of the tissue-specific transcript Tex19.1 served as a positive control for the Dnmt3b deletion (Figure S5f in Additional file 1, right panel) and the Tex19.1 promoter was hypomethylated in the absence of Dnmt3b ([18]; data not shown). Consistent with previously published data, *Dnmt1*^*−/−*^ MEFs also mis-expressed IAP elements and satellites (Figure S5g in Additional file 1) [18]. In summary, although *Lsh*^*−/−*^ and *Dnmt3b*^*−/−*^ mutant cells had similar HELP-seq methylation profiles at satellites and LINE-1, the *Dnmt3b*^*−/−*^ MEFs showed no significant expression changes at repeat elements, whereas Lsh mutant fibroblasts mis-expressed multiple discrete repeat classes.

### Lsh deletion leads to the appearance of anomalous Dnmt1 foci and accumulation of retroviral intracisternal A-particle protein

**Figure 6:**
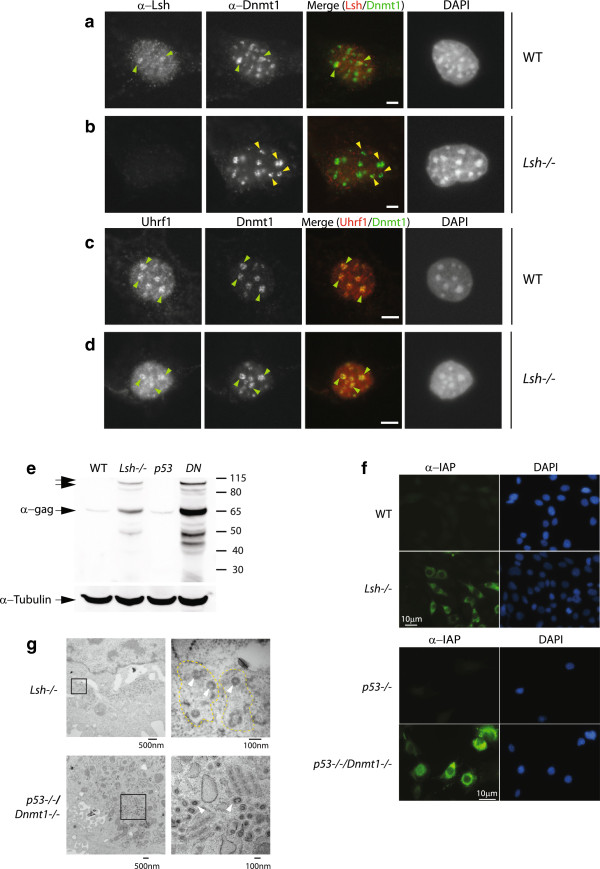
**Dnmt1 and Uhrf1 protein expression in Lsh**^**−/− **^**cells and activation of intracisternal A**-**particle protein in DNA hypomethylated cell types. (a)** Indirect immunofluorescence on WT fibroblasts using antibodies against Lsh and Dnmt1. Green arrowheads indicate co-localisation. Merge: Lsh and Dnmt1 pseudo-coloured red and green respectively. **(b)** Indirect immunofluorescence Lsh^−/−^ fibroblasts as in (a). Crescent-shaped disrupted Dnmt1 foci (yellow arrowheads). **(c)** Indirect immunofluorescence of WT fibroblasts using Uhrf1and Dnmt1 antibodies. Co-localisation of Uhrf1 and Dnmt1 (green arrowheads). Merge: Uhrf1 and Dnmt1 are pseudo-coloured red and green respectively. **(d)** Indirect immunofluorescence as in (c). Co-localisation between Uhrf1 and Dnmt1 (green arrowheads). In the merged panel, Uhrf1 is pseudo-coloured red and Dnmt1 is pseudo-coloured green. DNA is stained with DAPI. Scale bar = 5 μM. **(e)** Lanes: WT, *Lsh*^*−/−*^: western blots on WT and *Lsh*^*−/−*^ lysates probed with an IAP-*gag* specific antibody. Lanes: *p53*^*−/−*^, DN: western blots on *p53*^*−/−*^ and DN lysates probed with the IAP-*gag* specific antibody. Molecular weight of proteins is indicated in kDa on the right; prominent bands are annotated on the left. Higher mobility protein species (unannotated arrows) may represent IAP *gap-pro* or *gag-pro-pol* fusions. Lysate loading control: alpha-tubulin. **(f)** Indirect immunofluorescence on fixed cells using the same IAP-*gag* antibody as in (a). Left: WT and *Lsh*^*−/−*^ fixed cells. Right: *p53*^*−/−*^ and DN fixed cells. IAP staining was detected with an Alexa Fluor 488 secondary antibody yielding a green stain. Nuclei are counterstained blue with DAPI. Scale bar = 10 μM. **(g)** Transmission electron microscopy on *Lsh*^*−/−*^ and DN fixed cells. Left panel: lower magnification of subcellular structures; note the presence of multiple darker round structures. Boxed area is shown at higher magnification on the right. White arrows indicate examples of VLP. Hashed yellow area indicates endoplasmic reticulum cisternae. Scale bar = 500 nM (left) and 100 nM (right). DN; *p53−/−* | *Dnmt1−/−* double knockout MEFs.

One explanation for the similarity between the Lsh and Dnmt1 mutant repeat expression profiles (Figure 3 and Figure S5 in Additional file 1) is the possibility that the two proteins are spatially co-localised at heterochromatic foci in mouse nuclei. Previous work has demonstrated that Dnmt1 localisation in mouse cells is dynamic, and that it is located on heterochromatin (4′-6-diamidino-2-phenylindole (DAPI)-positive spots) during late S-phase [6]. Using immunofluorescence to detect the endogenous proteins, we observed overlap between Dnmt1 and Lsh punctate foci in WT fibroblasts, coincident with heterochromatic regions (DAPI staining) (Figure 6a, green arrowheads). Interestingly, Dnmt1 protein was present at heterochromatic foci in *Lsh*^*−/−*^ mutant fibroblasts, however some Dnmt1 foci (approximately 30%) displayed crescent-shaped patterns that may be indicative of heterochromatic disruption (Figure 6b, yellow arrowheads). Given the cooperative roles played by Uhrf1 (NP95) and Dnmt1 in DNA methylation deposition, we asked whether localisation of Uhrf1 was perturbed in the absence of Lsh. In agreement with previous observations from Sharif and colleagues, Dnmt1 and Uhfr1 were tightly coupled in S-phase nuclei (Figure 6c, green arrowheads) [7]. Notably, the majority of Uhfr1 protein tracked Dnmt1 localisation (or *vice versa*) in *Lsh*^*−/−*^ nuclei (Figure 6d), indicating that Lsh deletion does not dramatically affect the association between Uhrf1 and Dnmt1. These results suggest the Lsh protein is not required for the association between Dnmt1 and Uhrf1. The significant levels of methylation still present at major satellite repeats in *Lsh*^*−/−*^ cells may account for the continued association of the Dnmt1/Uhrf1 complex during late S-phase, while it is possible that the crescent-shaped patterns correspond to more hypomethylated satellites.

Approximately 1,000 full-length IAP sequences are present in the murine genome, hundreds of copies of which retain retrotransposon activity, rendering this class of elements one of the most active [48]. Phylogenetic analyses imply that murine IAPs are a derivative of an ancestral retrovirus that has reached the germline of a remote rodent ancestor subsequent to loss of the *env* gene; consistent with its atypical intracellular lifecycle. Importantly, only approximately 700 of the around 1,000 generic IAP copies are full-length, that is, contain intact LTRs, *gag*, *pro* and *pol* genes. Our DNA methylation, transcription and chromatin analyses highlight a considerable de-repression of IAP sequences in the absence of Lsh. However, in terms of the functional consequences of Lsh deletion, it is unclear whether IAP transcription results in the translation of functional IAP proteins. Immunoblotting experiments detected full-length IAP protein in both DNA hypomethylation mutant backgrounds (Figure 6e), which is consistent with the IAP transcriptional activation by RNA-seq and previously published findings [39].

Overexpression of retrocompetent IAP constructs into 293 T cells leads to accumulation of IAP VLP at the cisternae of the endoplasmic reticulum [49]. We examined the localisation of endogenous IAP protein in *Lsh*^*−/−*^ and *Dnmt1*^*−/−*^ fibroblasts by indirect immunofluorescence and, as illustrated in Figure 6f, IAP protein was largely excluded from nuclei and accumulated in aggregates proximal to the nuclear periphery; potentially co-localising to endoplasmic reticulum subcellular structures. We detected IAP accumulation in approximately 35% of *Lsh*^*−/−*^ cells, which may imply additional mechanisms of IAP regulation. Similar to Lsh mutant cells, *Dnmt1*^*−/−*^ fibroblasts (which were severely DNA hypomethylated) showed considerable levels of IAP protein aggregates adjacent to the nuclear periphery with exclusion from nuclei (Figure 6f). We viewed WT and *Lsh*^*−/−*^ mutant cells at higher resolution by transmission electron microscopy to detect the possible presence of VLPs that appear in the cisternae of the endoplasmic reticulum when IAP constructs are overexpressed in human cells [48]. We observed the presence of multiple VLPs (Figure 6g, top panel; white arrowheads) in *Lsh*^*−/−*^ cells within structures adjacent to the nuclear membrane resembling endoplasmic reticulum cisternae (Figure 6g; yellow outlines) and their absence from WT cells (data not shown). We also detected the presence of VLPs by transmission electron microscopy in most *Dnmt1*^*−/−*^ cells scored and their absence in corresponding control cells (Figure 6g, lower panel). We noted the two concentric shells surrounding an electron-lucent core within the VLPs [48]. Consistent with the absence of IAP transcription in *Dnmt3b*^*−/−*^ cells, we could not detect VLPs in this mutant (Figure S5h in Additional file 1).

It is not clear why IAP protein expression is more pronounced in the *Dnmt1*^*−/−*^ null background compared to the *Lsh*^*−/−*^ background, but this may be due to the relative severity of hypomethylation in these mutants or the coding capacity of the IAP transcripts de-repressed (Figure S2g,h in Additional file 1). The *Dnmt1*^*−/−*^ cells were established as MEFs from embryos and so may represent a hypersensitive developmental time point compared to terminally differentiated *Lsh*^*−/−*^ tail-tip fibroblasts. In summary, these experiments show that *Lsh*^*−/−*^ deletion leads to marked activation of repeat transcripts that in certain cases can result in the production and appropriate localisation of potentially harmful intracellular retroelement particles.

### The combinatorial histone code at repeat sequences is disrupted in DNA hypomethylated mutants

**Figure 7:**
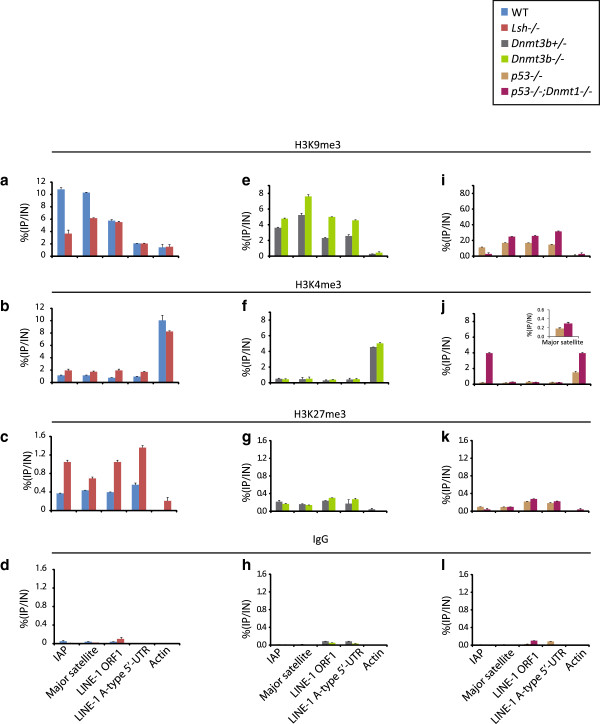
**Histone modification states at repetitive elements. (a**-**d)** ChIP of modified histone tails (H3K9me3, H3K4me3 and H3K27me3) followed by qPCR analysis in WT and *Lsh*^*−/−*^ fibroblasts at indicated repeat sequences and beta-actin. **(e**-**h)** ChIP of modified histone tails followed by qPCR analysis in *Dnmt3b*^*+/−*^ and *Dnmt3b*^*−/−*^ MEFs at indicated repeat sequences and beta-actin. **(i**-**l)** ChIP of modified histone tails followed by qPCR analysis in *p53*^−/−^ and *p53*^*−/−*^ |*Dnmt1*^*−/−*^ MEFs at indicated repeat sequences and beta-actin. Blue = WT fibroblasts; red = *Lsh*^*−/−*^ fibroblasts; green = *Dnmt3b*^*+/−*^ MEFs; light green = *Dnmt3b*^*−/−*^ MEFs; orange = *p53*^*−/−*^ MEFs; purple = *p53*^*−/−*^ |*Dnmt1*^*−/−*^ MEFs. Experiments represent triplicate analysis. Histone modification indicated above each plot. See Figure S6 in Additional file 1 for ChIP primer locations. Units are expressed as percentage enrichments that is, % (immunoprecipitated DNA/input DNA). Ig, immunoglobulin; IN, input DNA; IP, immunoprecipitated DNA.

A combination of post-translationally modified histone tails has been proposed as a code that ultimately impacts on transcription and chromatin compaction [50]. Previous studies have defined a profile of repressive histone lysine methylation states for the repetitive complement of the mouse epigenome [51]. Further observations in *Neurospora* imply that DNA methylation and repressive histone marks can act synergistically to repress parasitic DNA elements in eukaryotic genomes [52]. One reported histone defect in *Lsh*^*−/−*^ nuclei was gross changes in H3K4me2 (a mark associated with activation) localisation compared to WT cells [53]. Given our DNA methylation and transcription results, we used cross-linked and native chromatin immunoprecipitation (ChIP) to test if the chromatin landscape of specific repeat classes was altered for modified histones. To control for the specificity of immunoprecipitated DNA pulled down, we also carried out ChIP with normal immunoglobulin G, which had very low enrichments for these high-copy repeat classes (Figure 7d, h, l). In agreement with aberrant hypomethylation and transcription from major satellites and IAP retrotransposons in *Lsh*^*−/−*^ mutants, ChIP showed a decrease in H3K9me3 (repressive mark) at these regions (Figure 7a). Despite the hypomethylation at LINE-1 sequences, we did not detect any H3K9me3 changes at LINE-1s (either in LINE-1 ORF1 or the younger A-type LINE-1 5′UTR monomer) - this is consistent with the overall lack of LINE-1 de-repression (Figures 3c-f and 7a). We detected corresponding gains of H3K4me3 (active mark) at IAP, satellites and LINE-1 regions (Figure 7b). Bivalent chromatin is enriched in both H3K4me3 and H3K27me3 and is associated with poised CpG island (CGI) gene promoters in pluripotent cells, which are either destined for subsequent repression (loss of H3K4me3) or activation (loss of H3K27me3) [54]. Despite being from a low initial value, we detected gains of H3K27me3 enrichment in all repeat components examined (Figure 7c), implying inappropriate targeting of this modification to repeat elements in the absence of Lsh. While it is possible that unique repeat loci losing H3K9me3 are also modulated in terms of their H3K4me3 or H3K27me3 modification state, it is not clear if this occurs at all repeat loci. In summary, loss of H3K9me3 coupled to H3K4me3 gains (despite moderate accumulation of H3K27me3) correlated with robust transcription from IAP and satellite sequences in fibroblasts lacking Lsh. By contrast, LINE-1 sequences did not lose H3K9me3 - which appears to be incompatible with LINE-1 transcription.

We also carried out ChIP analyses on *Dnmt1*^*−/−*^ and *Dnmt3b*^*−/−*^ MEFs and their respective controls. Here we observed moderate gains in H3K9me3 enrichment in *Dnmt3b*^*−/−*^ cells at all repeats tested and no major changes in H3K4me3 and H3K27me3 levels (Figure 7e-h). Similar to *Dnmt3b*^*−/−*^, we observed slight gains in H3K9me3 at major satellites and LINE-1 sequences in *Dnmt1*^*−/−*^ cells. The notable exception to H3K9me3 gains was a loss of this mark at IAP sequences and a strong accumulation of H3K4me3 in the absence of Dnmt1, which correlated with the marked de-repression of IAP in Lsh and Dnmt1 mutants (Figure 7i-l). IAPs are likely to be co-regulated (at least in part) by Dnmt1 and Lsh as the epigenetic and chromatin changes at IAP regions in both mutants are similar. Surprisingly, although *Dnmt3b*^*−/−*^ cells are DNA hypomethylated, there were no major changes in repeat expression or chromatin state (aside from gains in H3K9me).

## Discussion

Of the numerous modifications linked with epigenetic transcription regulation, DNA methylation appears to be the most functionally apparent, that is, its presence at promoters is associated with a repressed state and hypomethylation is associated with a more transcriptionally permissive state [18]. The most striking examples of the dynamic nature of global DNA methylation are thought to occur during embryonic development. Upon fertilisation, the mouse paternal genome undergoes DNA methylation erasure followed by re-establishment of this epimark later in development; in addition, primordial germ cells are extensively reprogrammed to produce viable mature germ cells [55,56]. To re-establish global methylation in the early embryo, the *de novo* and maintenance methyltransferases must be targeted to the appropriate unmarked DNA loci. How this is achieved is not fully understood, but Uhrf1-dependent histone H3 ubiquitylation is required to target Dnmt1 to replication sites *in vitro*[57]. Another strong candidate for targeting *de novo* methylation is the putative chromatin remodeller Lsh, as its deletion leads to hypomethylation in surviving embryos and mice [10,58,59].

In this study we investigated the DNA methylation, transcription and chromatin states of repetitive elements in *Lsh*, *Dnmt1* and *Dnmt3b* mouse knockout cells. We employed HELP-seq for global DNA methylation profiling, which yields a quantitative measure of methylation at approximately 2.1 million CCGG sites (at the inner cytosine) throughout the mouse genome. This approach extends previous qualitative investigations [32] and obviates any inherent immunoprecipitation biases [60]. Primarily, our genome-wide approach clearly highlighted the specificity of methylation defects in the absence of Lsh. Moreover, our data support a model that Lsh recruits *de novo* methylation via Dnmt3b at certain repeat classes (LINE-1 and satellite), which are hypomethylated in *Dnmt3b*^*−/−*^ and *Lsh*^*−/−*^ cells. By contrast, the LTR class of repeats are generally less hypomethylated in *Dnmt3b*^*−/−*^ cells compared to *Lsh*^*−/−*^ cells. In addition, it is evident that while specific repeat classes are targeted (satellite DNA, LTR and LINE-1), others (for example, SINEs) appear to be largely unaffected. One question that arose from our results was the possibility that the repeat classes that escape hypomethylation in *Lsh*^*−/−*^ mutants are not methylated in WT fibroblasts. To test this, we parsed whole-genome reduced representation bisulfite data obtained from MEFs (with normal methylation patterns) by individual repeat class. We observed that only tRNA, low complexity and simple repeats were largely unmethylated compared to rRNA, SINE and DNA repeat classes, which were heavily methylated in WT cells (Figure S2g in Additional file 1). By high-throughput methylation analyses, we have shown that Lsh and Dnmt3b mutant cells are hypomethylated at multiple repetitive DNA compartments, most notably at satellites and LINEs. In the case of the LTR class of repeats, hypomethylation was more severe in the absence of Lsh than Dnmt3b (Figure 1f) and we propose that, in contrast to Lsh, Dnmt3b has a reduced role in the regulation of DNA methylation at LTRs.

RNA-seq has been used to survey the transcriptional impact upon deletion of three methyltransferases (Dnmt1, Dnmt3a and Dnmt3b) in pluripotent mouse ES cells [46]. Surprisingly, one conclusion from this study was that despite lacking three major methyltransferases and having severely hypomethylated DNA, the effect on repetitive element transcription was minimal. By contrast, deletion of the H3K9me3 histone methyltransferase SetDB1 or the associated binding partner KAP1 has been to shown to lead to the accumulation of ERV transcripts [46], thus demonstrating that different regulatory mechanisms exerted by DNA methylation exist between somatic cells and ES cells. We performed RNA-seq on embryos (E13.5; WT and *Lsh*^*−/−*^), MEFs (WT and *Dnmt1*^*−/−*^) and adult tail-tip fibroblasts (WT and *Lsh*^*−/−*^) to test the degree to which repetitive elements are transcriptionally sensitive to hypomethylation. Our investigations demonstrated that, unlike ES cells, both E13.5 and adult somatic mutants lacking components of the DNA methylation machinery are highly sensitive to hypomethylation at repeat sequences. This is unlikely to be caused by general deregulation of global transcription because genomic loci remote from the activated annotated repetitive elements are largely devoid of RNA-seq reads. We noted that, although LINE-1 sequences were hypomethylated, they retained transcriptional repression in the absence of Lsh, Dnmt3b or Dnmt1.

Interestingly, a short burst of strand-specific satellite transcription is coincident with the two-cell stage of mouse embryogenesis [26], and disruption of this process leads to improper chromocentre formation thereby impeding developmental progression. On the Lsh mutant background, transcription is initiated from both sense and antisense strands equally and *Lsh*^*−/−*^ mutant embryos appear to be karyotypically normal. Notably, repeat classes that do not show methylation changes by HELP-seq lack transcriptional activation in Lsh mutants. However, DNA hypomethylation in germ cells is linked to LINE-1 activation, although it is possible that LINE-1 chromatin signatures are different in these specialised cells [61]. The observed hypomethylation in *Lsh*^*−/−*^ E13.5 embryos and differentiated fibroblasts may be due to an ‘inherited’ DNA methylation defect that occurred during the global *de novo* methylation phase in early development. Thus, perturbation of Lsh in cells which have already passed through normal development and DNA methylation reprogramming phases may have no consequence. In our hands, depletion of Lsh in WT fibroblasts and ES cells by short hairpin RNA knockdown had no effect on global DNA methylation when compared to *Lsh*^*−/−*^ genomic DNA (Figure S7 in Additional file 1). This observation is in agreement with other studies where short hairpin RNA interference with Lsh levels in mouse cells did not alter methylation levels at the methyl-dependent gene *Rhox2* although it is abnormally hypomethylated in *Lsh*^*−/−*^ fibroblasts [29]. Similarly, over-expression (cytomegalovirus-promoter driven) of WT Lsh did not restore global methylation levels in *Lsh*^*−/−*^ fibroblasts as assayed by sensitivity to *Hpa*II and *Mae*II digestion (Figure S7e in Additional file 1). One interpretation of these observations is that there is a precise developmental window during which Lsh participates in *de novo* methylation targeting.

Examples of genes regulated solely by DNA methylation are rare. For example, DNA methylation exerts strong repressive effects in somatic cells on the lineage restricted gene Tex19.1 (and other germ-line genes) [18]. This example of regulation by one dominant epigenetic modification mechanism may be the exception rather than the rule; it is widely accepted that DNA methylation and chromatin states cooperate to establish or maintain silencing and define cellular identity [62,63]. Lsh has been identified as a minor protein partner of the repressive chromatin modifying complex Polycomb repressor complex 1, which targets developmentally regulated homeobox genes as well as being required to repress satellite transcription in the zygote [64]. In ES cells, Lsh is required for targeting Dnmt3b to the Oct4 promoter during differentiation [30]. Guided by our *a priori* HELP-seq and RNA-seq data, we used ChIP to look at multiple histone tail modifications at repeat sequences, which indicated two general trends in *Lsh*^*−/−*^ cells: gains of the activating mark H3K4me3 and, more surprisingly, significant accumulation of H3K27me3. By contrast, the classical heterochromatin mark H3K9me3 was only depleted from repeat classes that were actively transcribed, that is, LTR and satellites. Our investigations imply that DNA methylation loss in the absence of Lsh is not necessarily sufficient to support transcription and that loss of H3K9me3 is also required. Moreover, although the *Dnmt3b*^*−/−*^ genome is hypomethylated, this is not accompanied by genome-wide transcription or indeed alterations in chromatin modification state at repeats. Whether Dnmt3b and Dnmt1 (and indeed Lsh) associate with different chromatin modifying protein partners, thereby explaining the dissimilarity (at the molecular level) between their respective disruptions, remains unknown. A previous report highlighted the possibility that Lsh recruits the histone methyltransferase G9a to gene promoters thereby reinforcing silencing [29]. Data mining and analysis of a G9a knockout MEF expression microarray dataset revealed very few expression changes at single copy genes or probes that overlap annotated repeat elements (data not shown) [22,65]. Due to the repressive roles played by G9a at euchromatic loci [29], it is possible that Lsh cooperates with different heterochromatic histone methyltransferases at repetitive DNA loci to maintain silencing at these regions.

**Figure 8:**
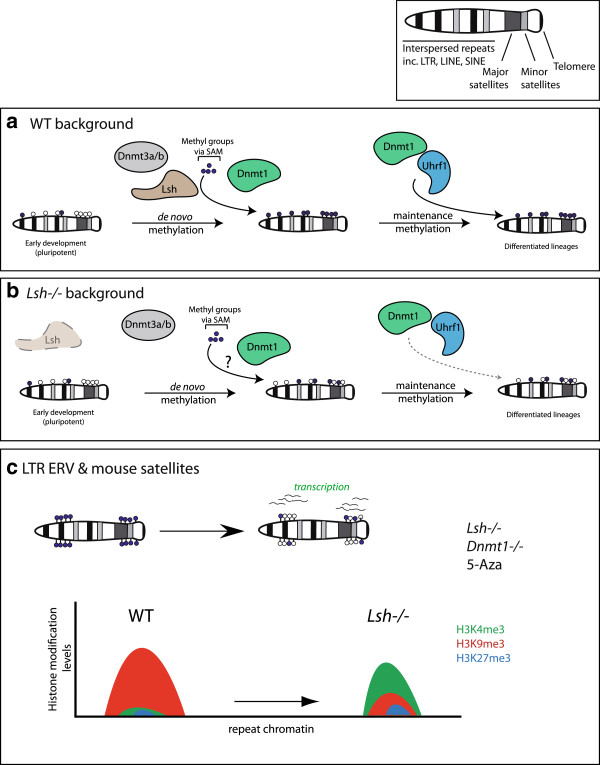
**Putative model for DNA methylation**-**dependent repeat regulation by Lsh during embryonic development. (a)** WT background: the early pluripotent embryo is globally hypomethylated compared to the heavily methylated one-cell zygote (which experiences a wave of DNA demethylation shortly after fertilisation). Subsequently, a wave of *de novo* global DNA methylation mediated by Dnmt3a and Dnmt3b in cooperation with Lsh (and perhaps in part Dnmt1/UHRF1) occurs, thereby re-imposing methylation. At later embryonic stages, Dnmt1 and UHRF1 cooperate to maintain stable DNA methylation patterns as lineages differentiate. **(b)** Lsh mutant background: in the absence of Lsh, *de novo* methylation may be perturbed in some cases. Later stage Lsh mutant embryos are presumably unable to target the maintenance methyltransferase Dnmt1 to specific repeat compartments (including LTR-ERV, LINE-1 and satellites), leading to the hypomethylation phenotypes observed in these mutants. **(c)** Lsh and Dnmt1 are functionally both required to regulate LTR-ERVs (independent of Dnmt3b) in maintaining a highly DNA methylated and H3K9me3 associated chromatin state (left). In the absence of Lsh or Dnmt1, LTR-ERVs have reduced DNA methylation and H3K9me3 levels, are actively transcribed and acquire the activation associated histone modification H3K4me3 (right). SAM: S-adenosyl methionine.

Our observations raise the possibility that Lsh is specifically required during the global *de novo* methylation phase (post-implantation) in embryo development to target methylation (and histone repressive marks) at specific repeat classes, but it is not required to maintain a fully methylated genome in differentiated cells (see model in Figure 8). It is not yet clear why LINE-1s behave differently to other hypomethylated repetitive DNA classes. In stem cells lacking either Eset (Setdb1) or Hdac1, LINE-1 activation is minimal [24,46,66]. In the Lsh null background, one possibility is that additional repressive activities exist that permit LINE-1 to tolerate CpG methylation loss and H3K4me3 gains and yet remain silent. The genomic organisation of LINE-1s differ from LTR-ERVs: LINE-1s lack a conserved CGI promoter and distinct LINE-1s are associated with different promoter arrangements [67]. Many mouse LINE-1s are inactive due to the truncation of the 5′-UTR as a consequence of poor LINE-1 reverse transcriptase processivity or when target site sequences can base-pair with the integrating LINE-1 sequence [67]. The sequence arrangement of the 5′-UTR in retrocompetent full-length young LINE-1s may add a level of regulatory control that is distinct from LTR-ERVs and satellite DNA [49]. The IAP LTR element typically lacks an *env* gene and has a mutated *gag* gene, which ensures that mature VLPs are unable to exit the host cell [49]. Immunohistochemistry experiments show that hypomethylated DNA induced by Lsh deletion (and Dnmt1 deletion) is not attributable to transcriptional noise from repeat DNA fragments - there is functional production of full-length IAP protein in knockout cells. Moreover, the IAP is excluded from the nucleus in methylation mutants and instead accumulates in the cytoplasm as a VLP.

Collectively, our findings emphasise that there is appropriate programmed deposition and maintenance of DNA methylation during development. A striking feature of the *Lsh*^*−/−*^ chromatin landscape is the acquisition of the H3K27me3 modification at repeat DNA, revealing an intriguing similarity to the *Dnmt1*^*−/−*^ mutant in which H3K27me3 is redistributed from Polycomb target genes, leading to their activation and silencing at genes that attract *de novo* H3K27me3 deposition [33]. Importantly, limited H3K27me3 enrichment at hypomethylated sites in *Lsh*^*−/−*^ cells is not a significant barrier to transcription of IAPs and major satellite, implying that normal DNA methylation patterns is the dominant repressive mechanism at these loci. It will be of great interest to investigate if there is a similar global redistribution H3K27me3 from unique genes in *Lsh*^*−/−*^ cells leading to their activation and perhaps silencing at hypomethylated genes that attract *de novo* H3K27me3 deposition.

## Conclusions

We have highlighted the complexity of somatic cell repeat DNA compartment regulation, as exemplified by Lsh-mediated regulation of IAP LTRs - this contrasts with the maintenance of IAP LTR silencing in *Dnmt3b*^*−/−*^ cells. Furthermore, while Lsh is implicated in the regulation of single copy gene promoters via Dnmt3b recruitment, our results support a possible role for Lsh in targeting all DNA methyltransferases to IAP LTRs and satellite sequences during development. Without this interaction, silencing cannot be initiated at these repeats and subsequently maintained in somatic cell lineages (Figure 8). We propose that hypomethylation of LINE-1s is not sufficient for their activation in fibroblasts and that tissue-type and chromatin states are potential determinants of LINE-1 silencing. One implication of this study is the possibility that a germline Lsh mutation (and indeed Dnmt mutations) may contribute to the hypomethylation phenotype observed in some human cancers [27]. Currently, the kinetics of repeat hypomethylation (and repeat transcription) in the *Lsh*^*−/−*^ background during early (pre-E13.5) embryonic development has yet to be reported. Does this defect initiate during the post-implantation or gamete *de novo* methylation phases? A conditional Lsh knockout embryo may clarify these developmental questions whereas a tissue-specific conditional Lsh knockout line may permit further dissection of the Lsh phenotype.

## Materials and methods

### Cells and embryos

Transformed (SV40) WT and *Lsh*^*−/−*^ fibroblasts isolated from mouse tail-tips were provided by Professor Robert Arceci (Phoenix Children’s Hospital, Phoenix, AZ, USA). Heterozygous and homozygous Dnmt3b mouse embryonic fibroblasts were SV40 large T-antigen transformed by transfection with an SV40 construct in a pBlueScript backbone followed by the 3 T3 protocol to select for transformants [68]. p53 and p53/Dnmt1 mutant mouse embryonic fibroblasts have been previously described [18,33]. All fibroblasts were cultured in 89% Dulbecco’s modified Eagle’s medium, 10% foetal calf serum and 1% penicillin streptomycin. Tissue from heterozygous and homozygous *Lsh* embryos was staged at E13.5, homogenised and snap frozen.

### HELP-seq and methylation analyses

HELP-seq was performed as previously described [32] and sequencing was performed on the HiSeq platform (Illumina, San Diego, CA, USA). Libraries were prepared from WT fibroblasts, *Lsh*^*−/−*^ fibroblasts, Dnmt3b^+/−^ MEFs and *Dnmt3b*^*−/−*^ MEFs. All downstream analyses were performed using the automated WASP pipeline at Einstein College of Medicine [35]. MeDIP was performed as previously reported [18]. Bisulfite conversions were performed using the EZ-DNA methylation gold kit (ZymoResearch, Irvine, CA, USA) and products were cloned into pGEM-T Easy (Promega, Madison, WI, USA) and plasmids were sequenced using BigDyev3.1 chemistry (Life Technologies, Grand Island, NY, USA). Bisulfite sequencing DNA methylation analysis was used to calculate percentage methylation in bisulfite sequencing clones [69]. To analyse unique repeat sequences in our HELP-seq libraries, we developed a custom pipeline as follows: raw HELP-seq *HpaII* library reads were mapped to the genome using Bowtie and multiple-copy sequences were excluded. Custom Perl scripts were generated to overlap unique *HpaII* reads with the mouse (build mm9) repeatmasker annotation file [70]. Non repeat-overlapping reads were discarded before counting reads in all repeat classes in driver and tester cell lines. Using the Text-NSP-1.03 CPAN package [71], 2 × 2 contingency odds ratios tables were produced. Finally, statistical testing between tester and driver lines was carried out using Fisher’s exact tests and Bonferroni correction for multiple testing.

### RNA-seq and analyses

Paired-end strand-specific mRNA-seq libraries were prepared using the dUTP method and sequenced using the HiSeq instrument (BaseClear, Leiden, Holland). Samples sequenced were WT fibroblasts, *Lsh*^*−/−*^ fibroblasts, WT E13.5 embryos and *Lsh*^*−/−*^ E13.5 embryos. Reads were mapped to the mouse genome (mm9) using TopHat v1.4.0 on a local Galaxy Server with the following settings: -p 4 -r 24 -a 8 -m 0 -i 70 -I 500000 -g 20 --library-type fr-firststrand --no-novel-indels --no-coverage-search --no-closure-search --mate-std-dev 27 --initial-read-mismatches 0 --segment-mismatches 0 -segment. A custom Galaxy pipeline was generated to filter for unique mRNA-seq reads from all libraries and to inner join output reads to the repeatmasker (mm9) annotation. Finally, the EdgeR package [45] was used to perform repeat element mRNA-seq differential analysis in the R programming environment [44].

### Chromatin methods

ChIP was performed using two methods, native-ChIP and cross-linked ChIP, performed as described previously [18,72]. For native ChIP, native chromatin was prepared from cell nuclei in NBR buffer (85 mM NaCl, 5.5% sucrose, 10 mM Tris, 3 mM MgCl_2_, 1.5 mM CaCl_2_, 0.2 mM phenylmethylsulfonyl fluoride, 1 mM dithiothreitol). Nuclei were treated with micrococcal nuclease (Worthington, Lakewood, NJ, USA) to generate mono-, di and tri-nucleosomes and chromatin was released overnight at 4°C. Immunoprecipitation was carried out with antibodies specific to H3K4me3 (07473, EMD Millipore, Billerica, MA, USA), H3K9me3 (ab8898, Abcam, Cambridge, UK), H3K27me3 (07449, Millipore) or rabbit immunoglobulin G (sc-2027, Santa Cruz Biotech, Dallas, TX, USA), followed by antibody-optimised washes. Samples from native ChIP were purified through affinity columns (Qiagen, Valencia, CA, USA) and quantified by qPCR using primers shown in Figure S6 in Additional file 1. For cross-linked ChIP, cells were cross-linked in 1% formaldehyde at room temperature for 10 min and extracts were sonicated in 1% SDS, 10 mM EDTA and 50 mM Tris–HCl (pH 8.0) and diluted 10-fold (supplemented to a final concentration of 1% Triton X-100) for immunoprecipitation for 3 h at 4°C. Antibodies used were as for native ChIP. ChIP and input chromatin was de-cross-linked at 65°C for 6 h, and then treated with RNaseA (Roche Diagnostics, West Sussex, UK) at 37°C for 1 h, then with Proteinase K at 55°C for 2 h. DNAs were purified using the QIAquick PCR purification kit (Qiagen). Real-time PCR analysis was carried on the LightCycler 480 System using SYBR Green Master mix (Roche) and oligonucleotides that are described in Table S1 in Additional file 1.

### Western and immunofluorescence analyses and transmission electron microscopy

Total cellular extracts were prepared directly from trypsinised cells using laemmli sample buffer or nuclear extracts were prepared using the Dignam method [73]. Protein concentrations were measured using the Bradford assay and 30 to 50 μg protein were resolved per lane on pre-cast NuPAGE Bis-Tris polyacrylamide gels (Life Technologies). Proteins were transferred to nitrocellulose, blocked with Western Block Reagent (Roche) diluted 1:40 with phosphate-buffered saline, and incubated with antibodies for either 1 h at room temperature or overnight at 4°C. Antibodies used: Lsh, #11955-1-AP (ProteinTech, Chicago, IL, USA); IAP-gag (Bryan Cullen, Duke, NC, USA); 5-methylcytidine, #BI-MECY-0500 (Eurogentec, Southampton, UK); single-stranded DNA, #JP18731, (Demeditec Diagnostics, Kiel-Wellsee, Germany).

For immunofluorescence, cells were grown on multichamber slides and fixed in paraformaldehyde. Immunostaining was performed using standard methods and antibodies used were the same as for immunofluorescence. For nuclear protein immunofluorescence, cells were incubated on ice in CSK buffer (100 mM NaCl, 300 mM sucrose, 3 mM MgCl_2_, 10 mM PIPES, pH6.8) for one minute prior to paraformaldehyde fixing. Additional antibodies included Lsh (#11955-1-AP, Protein Tech), Dnmt1 (#sc-10221, Santa Cruz Biotech) and Uhrf1 (#sc-98817, Santa Cruz Biotech).

For microscopy, the imaging system comprised a Coolsnap HQ CCD camera (Photometrics Ltd, Tucson, AZ, USA), Zeiss Axioskop II fluorescence microscope with Plan-neofluar objectives, a 100 W Hg source (Carl Zeiss, Welwyn Garden City, UK) and Chroma #89014 single emission filters (Chroma Technology Corp., Rockingham, VT, USA) with the excitation and emission filters installed in motorised filter wheels (Ludl Electronic Products, Hawthorne, NY, USA). Image capture and analysis were performed using in-house scripts written for IPLab Spectrum (Scanalytics Corp., Fairfax, VA, USA).

Transmission electron microscopy was performed using standard procedures. Cells were fixed in 2% glutaraldehyde (TAAB Laboratory Equipment, Aldermaston, UK) in sodium cacodylate buffer at 4°C overnight. Fixed cells were pelleted between reagent incubations at 3000 rpm for 1 min, decanted, and the next solution added. Cells were dehydrated using graded acetone at 25%, 50%, 75% (30mins each) and 100% (twice for 60 min). Cells were impregnated with 25% resin in acetone, 50% resin in acetone, 75% resin in acetone (60 min each) then 100% resin (TAAB Laboratory Equipment) for a minimum of three changes over 24 h. Embedding was carried out in 100% resin at 60°C for 24 h followed by sectioning into semi-thin survey sections of 0.5 μm and staining with 1% toluidine blue in 1% borax. Ultrathin sections (approximately 70 nm) were then cut using a diamond knife on a RMC MT-XL ultramicrotome (RMC Products, Tucson, AZ, USA). The sections were stretched with chloroform to eliminate compression and mounted on Pioloform-filmed copper grids. Staining reagents were 2% aqueous uranyl acetate and lead citrate (Leica, Allendale, NJ, USA). The grids were examined using a Philips CM 100 Compustage (FEI) Transmission Electron Microscope and digital images were collected using an AMT CCD camera (Deben UK Ltd., Suffolk, UK).

### Data access

Raw and processed data were deposited into the Gene Expression Omnibus and is freely available [GEO:GSE52479]. Other datasets utilised in this study are as follows. DNA methylation in *Dnmt1*^*+/+*^ and *Dnmt1*^*−/−*^ MEFs [GEO:GSE44278]: [GEO:GSM1090110] - *Dnmt1*^*+/+*^ MEFs (Bisulfite-seq); [GEO:GSM1090111] - *Dnmt1*^*−/−*^ MEF (Bisulfite-seq). RNA-seq in *Dnmt1*^*+/+*^ and *Dnmt1*^*−/−*^ MEFs [GEO:GSE44277]: [GEO:GSM1081738- GEO:GSM1081740] - *Dnmt1*^*+/+*^ MEF - (Replicates 1–3); [GEO:GSM1081741- GSM1081743] - *Dnmt1*^*−/−*^ MEF - (Replicates 1–3). RNA-seq in WT and triple knockout DNA methyltransferase ES cells [GEO:GSE29413]: [GEO:GSM727427] - J1 WT (mRNA-seq); [GEO:GSM727428] - J1 DMNT TKO (mRNA-seq) [50].

## Abbreviations

ChIP: chromatin immunoprecipitation; CpG: palindromic CG DNA dinucleotide; CGI: CpG island; DAPI: 4′,6-diamidino-2-phenylindole; DN: *p53−/−* | *Dnmt1−/−* double knockout MEFs; Dnmt: DNA methyltransferase; ERV: endogenous retrovirus; ES cells: embryonic stem cells; GAPDH: glyceraldehyde 3-phosphate dehydrogenase; H3K27me3: histone H3 lysine 27 trimethylation; H3K4me2: histone H3 lysine 4 dimethylation; H3K4me3: histone H3 lysine 4 trimethylation; H3K9me3: histone H3 lysine 9 trimethylation; HELP-seq: HpaII tiny fragment enrichment by ligation-mediated PCR followed by tag sequencing; IAP: intracisternal A-type particle; LINE-1: long interspersed nuclear elements; LTR: long terminal repeat; MeDIP: methylated DNA immunoprecipitation using 5meC antibodies; MEF: mouse embryonic fibroblast; ORF1: open reading frame 1; qPCR: quantitative polymerase chain reaction; qRT-PCR: quantitative reverse transcription polymerase chain reaction; RNA-seq: messenger RNA sequencing; SINE: short interspersed nuclear elements; TKO: triple knockout; UTR: untranslated region; VLP: viral-like particle; WT: wild type.

## Competing interests

The authors declare that they have no competing interests.

## Authors’ contributions

DSD, IRA and RRM conceived and designed the experiments. DSD performed the experiments with HC performing the immunofluorescence and TD the transmission electron microscopy. DSD, MS and JG performed the HELP-seq library preparation. DSD, RRM and IRA analysed the data. RA, CS and IRA contributed reagents, materials and analysis tools. DSD and RRM wrote the paper. All authors read and approved the final manuscript.

## Authors’ information

IRA and RRM are joint senior authors.

## Supplementary Material

Additional file 1 Included are seven supplemental figures (Figures S1 to S7) and one supplemental table (Table S1). Click here for file
