# Supplementary material for: Lsh regulates LTR retrotransposon repression independently of Dnmt3b function
Source: Genome Biol. 2013 Dec 24;14(12):R146. doi: 10.1186/gb-2013-14-12-r146 (PMC4054100; doi:10.1186/gb-2013-14-12-r146)
Supplement: Additional file 1 — Included are seven supplemental figures (Figures S1 to S7) and one supplemental table (Table S1). [file gb-2013-14-12-r146-S1.pdf]

## Additional_file_1 as PDF: Legends

**Figure S1.** No changes in methyltransferase levels in the absence of Lsh and hypomethylation of Lsh nuclei. **a)** Left panel: indirect immunofluorescence on fixed cells using a 5-methylcytosine (left) antibody and a single-stranded DNA (right) antibody. 5meC staining was detected with an Alexa Fluor 594 secondary antibody yielding a red stain and single-stranded DNA with an Alexa Fluor 488 secondary yielding a green stain. Note the absence of multiple condensed heterochromatic foci in *Lsh*$^{-/-}$ nuclei compared to wildtype nuclei indicated by white dashed circles. Right panel: indirect immunofluorescence on fixed cells using an Lsh antibody (left) and DAPI stained DNA (right). Lsh staining was detected with an Alexa Fluor 488 secondary antibody yielding a green stain. Note the absence of Lsh nuclear staining in Lsh$^{-/-}$ cells compared to wildtype cells. Scale bar = 10µM. **b)** Wild type and *Lsh-/-* genomic DNA was mock-digested and digested with Msp I, Hpa II and MaeII, which reveals normal methylation digestion profiles in wild type cells. In *Lsh-/-* cells note the small digestion fragments (black arrowheads) with Mae II digestion; indicative of hypomethylation. The Mae II digest were also run side by side for direct comparison. **c**) Graph indicating no differences in transcript levels (microarray) for DNA methyltransferases in wild type and *Lsh-/-* fibroblasts.

**Figure S2.** HELP-seq Additional file. **a)** Example of HELP-seq data in Dnmt3b knockout system. Left: a HELP-seq browser track showing mouse chr2:95100568-951000802 (genomic build mm9) and loss of methylation at the indicated (asterisk) LINE-1 element as in Figure 1. **b)** HELP-seq browser track showing mouse chr15:31097720-31097931 (genomic build mm9) and no change in DNA methylation at indicated (asterisk) genomic locus. **c)** Bioinformatics pipeline scheme used to analyse HELP-seq repeat element data. **d)** Analysis of genome-wide HELP-seq DNA methylation difference reproducibility in biological replicate libraries at repeat classes in WT & *Lsh*$^{-/-}$ cell line pair. Compare the OR profile for compiled replicates 2 and 3 (shown here) and replicate 1 shown in Figure 1. Scale is calculated odds-ratio (OR; Additional file 1: Figure S2). OR>1 hypomethylation in L*sh*$^{-/-}$ indicated by green arrow; OR<1 hypermethylation in *Lsh*$^{-/-}$ indicated by red arrow*.* **e)** Table of read counts and statistics for HELP-seq replicates 2 and 3 as shown above in S2d. **f)** Bisulfite sequencing pattern at mouse IAP LTR (left) and IAP *gag* gene (right) in heterozygous and homozygous Dnmt3b MEFs. Percentage methylation and cell types indicated. Black square = methylated; white square = unmethylated; red square = non-consensus CpG. See Additional file 1: Figure S6 for bisulfite primer locations. **g)** RRBS data mined for methylation levels at indicated repeat classes in *p53*$^{-/-}$ MEFs . **h)** RRBS data mined for methylation levels at indicated repeat classes in *p53*$^{-/-}$ |*Dnmt1*$^{-/-}$ MEFs .

**Figure S3.** Differential RNA-seq analysis of mouse IAP and LINE-1 in DNA methylation mutants. **a)** 10kb downstream IAP LTR probability density function plotted against fold change in indicated cell types. **b)** 10kb downstream non-IAP LTR fold-change plotted against

probability density function in indicated cell types. Note the lack of RNA-seq read changes between paired mutants and wildtype cells at regions 10kb downstream of annotated IAP LTRs (a) and non-IAP LTRs (b). Vertical lines indicate -5 and +5 $log_2$-fold changes. Scales: x-axis is $log_2$-fold change RNA expression; y-axis is probability density function (P.D.F.). **c)** Overlap between misexpressed LTRs in DNA hypomethylated cell types. Numbers of unique LTRs misexpressed in each mutant colour-coded: Lsh (blue) and DN (green). Interestingly, there is greater overlap in misexpression between non-IAP LTRs than IAP LTRs in Lsh and DN datasets. **d)** Boxplot of spread of distance between unique LTRs which change in expression in Lsh and DN datasets and the nearest annotated gene. Lsh IAPs are generally further from genes than the genome average and DN IAPs. In fact, DN IAPs are nearer to genes than the genome average. In contrast, Lsh and DN non-IAP LTRs are closer to neighbouring genes tan the gnome average. **e)** To determine if upregulated IAP LTRs or non-IAP LTRs are evolutionarily conserved we compared our LTRs to the published locations of all mouse IAPLTR1_Mm LTRs (n=1390) [77]. The generated LTR dataset was computationally divided into three distinct clades. Using BedTools we overlapped our LTRs with the published dataset and determined which clade our groups of LTRs belong to. Using chi-squared statistical tests, we found no significant difference between our LTR groups and the published LTR dataset (p>0.1 in all comparisons). X-axis is $log_2$-fold change in repeat RNA expression; y-axis is probability density function (P.D.F.).

**Figure S4.** DNA hypomethylation and expression of LINE-1s are poorly correlated in *Lsh*$^{-/-}$ mutants. Plot of RNA-seq expression changes versus HELP-seq DNA methylation changes in *Lsh*$^{-/-}$ versus WT cells. LINE-1s plotted are indicated in colour (yellow=LINE-1s that were significantly differentially expressed as shown in Figure 4; grey = all LINE-1s including non-significant elements which were present in both RNA-seq and HELP-seq datasets). Significant (and non-significant) differences detected using EdgeR in RNA-seq reads sequenced between Lsh$^{-/-}$ and WT cells are plotted against significant changes (Fisher's t-test and Benjamini-Hochberg multiple testing) in HELP-seq reads. Vertical dashed lines indicate -5 and +5 RNA-seq $log_2$-fold change thresholds and horizontal dashed lines indicates -1 and +1 $log_2$-fold change HELP-seq threshold. Note the pattern of expression: majority of LINE-1 are unchanged, a fraction are either upregulated or downregulated. However, many downregulated LINE-1s are hypomethylated, many upregulated LINE-1s are hypermethylated and *vice versa*.

**Figure S5.** Validation of repeat RNA-seq data by qRTPCR on embryonic *Lsh+/-* and *Lsh*$^{-/-}$ E13.5 isolates and strand-specific major satellite expression analysis. **a)** qRTPCR expression of IAP sequences in *Lsh+/-* and *Lsh*$^{-/-}$ E13.5 embryos showing major transcriptional derepression at IAP-*gag* and IAPEz-int. **b)** Similar to **(a)** marked derepression of mouse major satellite transcripts. **c)** SINE derived sequences show low expression in mutant embryos (note scale). **d)** LINE-1 5'UTR and ORF1 transcripts show little derepression in mutant embryos. **e)** GAPDH expression loading control for RNA samples analysed showing similar levels of this constitutive transcript. –rt shows undetectable signal in the absence of

reverse transcriptase. WT samples= red; *Lsh*$^{-/-}$ samples = orange. **f)** Strand-specific qRTPCR showed no preference for sense or antisense transcription in the absence of Lsh. All experiments are in triplicate. Expression level units are arbitrary and are all normalised to GAPDH expression levels. Also shown is Tex19.1 mis-expression in *Dnmt3b-/-* and *Dnmt1-/-* (DN) cells. **g)** Indicated repeat expression levels in *p53*$^{-/-}$ and *p53*$^{-/-}$|*Dnmt1*$^{-/-}$ MEFs. grey=*p53*$^{-/-}$ MEFs (p53); red=*p53*$^{-/-}$|*Dnmt1*$^{-/-}$ MEFs (DN). Experiments represent triplicate analysis. See Additional file 1: Figure S6 for qRTPCR primer locations. Expression level units are arbitrary (arbitrary expression units A.E.U.) and are all normalised to GAPDH expression levels. **h**) The TEM for *Dnmt3b-/-* cells does not detect VLPs.

**Figure S6.** Bisulfite, qRTPCR and ChIP primer maps on repeats. **a)** Bisulfite primers on major satellites, minor satellites and the IAP LTR are depicted. Black and white triangle pairs indicate primer pairs. Vertical blue lines indicate locations of CpG dinucleotides. Product sizes are indicated in base pairs. **b)** qRTPCR primers for expression analysis on IAP LTRs, major satellites, SINE B1, LINE-1 and Gapdh are depicted. Black and white triangle pairs indicate primer pairs. **c)** ChIP primers on major satellites, IAP LTRs, LINE-1 and beta-actin are depicted. Black and white triangle pairs indicate primer pairs.

**Figure S7.** Knockdown of Lsh does not induce hypomethylation in terminally differentiated tailtip fibroblasts nor embryonic stem cells**. a)** Lentiviral infection (#sh44 and #sh48) of WT tailtip fibroblasts reduces Lsh protein levels by 60-80% compared to non-mammalian target sequence (shNM). Extract dilutions were blotted and probed with Lsh and tubulin antibodies. **b)** Densitometry of western blots using ImageJ software. **c)** Lsh-depleted fibroblast genomic DNA retains normal methylation levels. Genomic DNA digested with MaeII reveals normal methylation digestion profiles in sh44 and sh48 DNA compared to shNM DNA. Lsh$^{-/-}$ genomic DNA is a positive control for hypomethylation – see small digestion fragments (arrowheads). **d)** IAP is not upregulated in Lsh knockdown fibroblasts by qRT-PCR – note similar levels of IAP expression in Lsh knockdowns ( sh44 & sh48) compared to shNM. **e)** Wildtype and mutant Lsh (ATPase mutant) do not rescue hypomethylation in Lsh$^{-/-}$ MEFs. Lsh$^{-/-}$ cells were transfected for 72 hours with GFP, GFP-Lsh or GFP-Lsh$^{ATPase}$ and genomic DNA isolations (including WT MEF DNA for comparison) were digested with MaeII or HpaII - note presence of small DNA digestion fragments (arrowheads) in Lsh cells transfected with either Lsh or Lsh$^{ATPase}$. **f)** Lsh-depleted ES cell genomic DNA retains normal methylation levels. Genomic DNA was digested with MaeII, revealing normal methylation digestion profiles in sh44 and sh48 DNA compared to shNM DNA. **g)** Clonal ES cells from anti-Lsh lentiviral infections are depleted for Lsh protein. Western blotting shows marked reduction in Lsh protein comparing five control ES clones (shNM) against six Lsh-depleted ES clones (sh44). Blots were probed as in (a). **h)** Lsh-depleted ES clone genomic DNA retains normal methylation levels. Genomic DNA was digested with Hpa II or Mae II revealing normal methylation digestion profiles in sh44 genomic DNA compared to shNM genomic DNA

# Supp S1

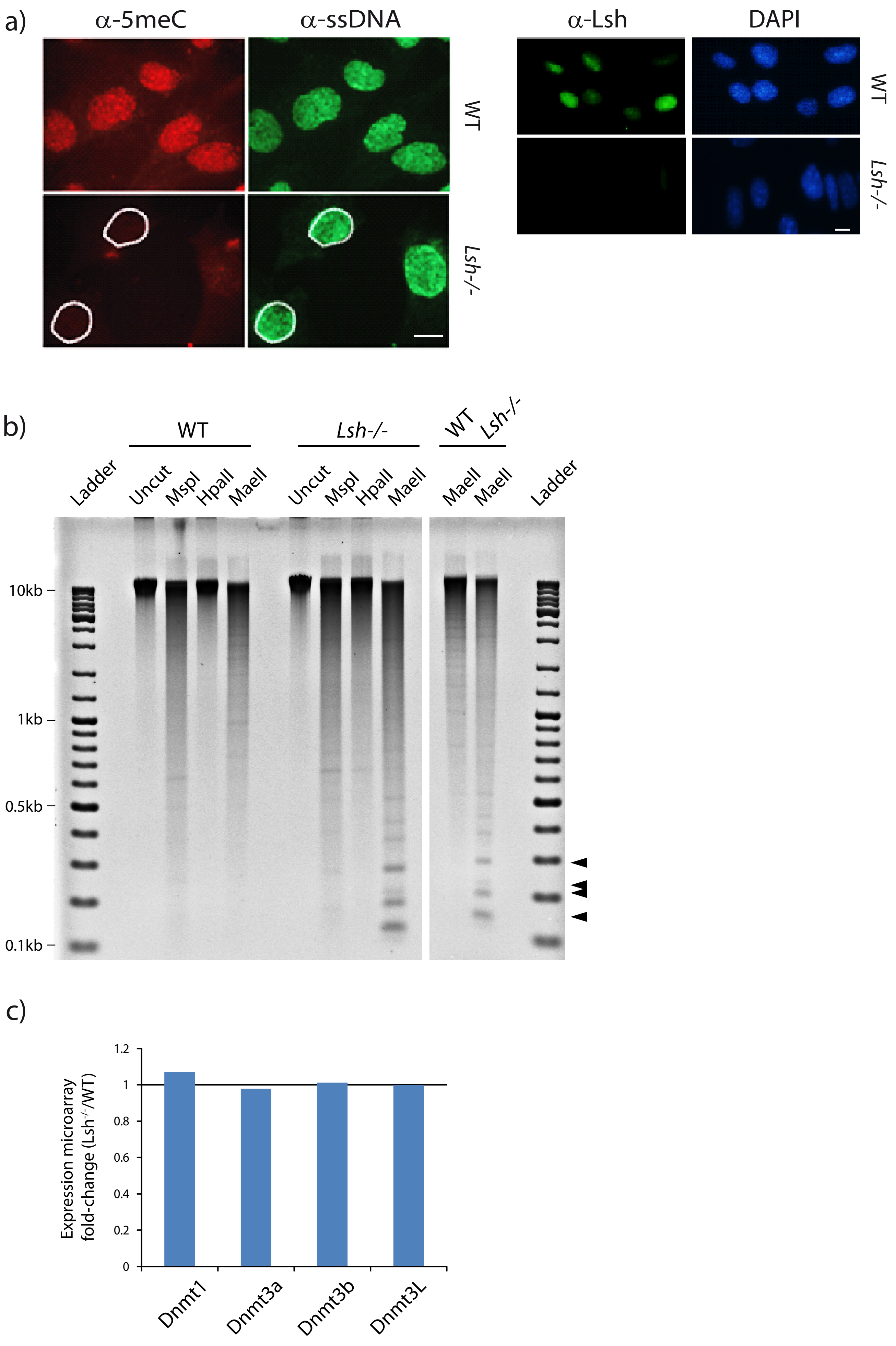

a)
α-5meC
α-ssDNA
α-Lsh
DAPI
WT
Lsh-/-
WT
Lsh-/-
b)
WT
Lsh-/-
WT
Lsh-/-
Ladder
Uncut
MspI
HpaII
MaeII
Uncut
MspI
HpaII
MaeII
MaeII
MaeII
Ladder
10kb
1kb
0.5kb
0.1kb
c)
Expression microarray fold-change (Lsh-/-/WT)
1.2
1
0.8
0.6
0.4
0.2
0
Dnmt1
Dnmt3a
Dnmt3b
Dnmt3L

# Supp S2

a)

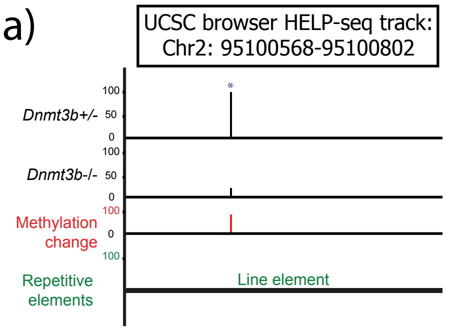


b)

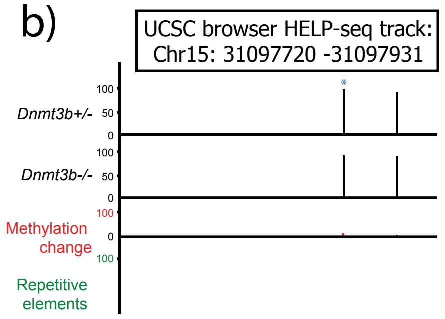


c)

<u>Repeat genomic methylation scheme</u>

i. HELP HpaII library reads mapped with Bowtie
ii. Repeatmasker annotated overlapping reads retained
iii. Reads counts compared between driver line (WT) and tester line (Lsh):
a) Fishers Exact t.testing
b) 2x2 contingency table odds-ratios (OR)
c) Bonferroni multiple testing correction

d)

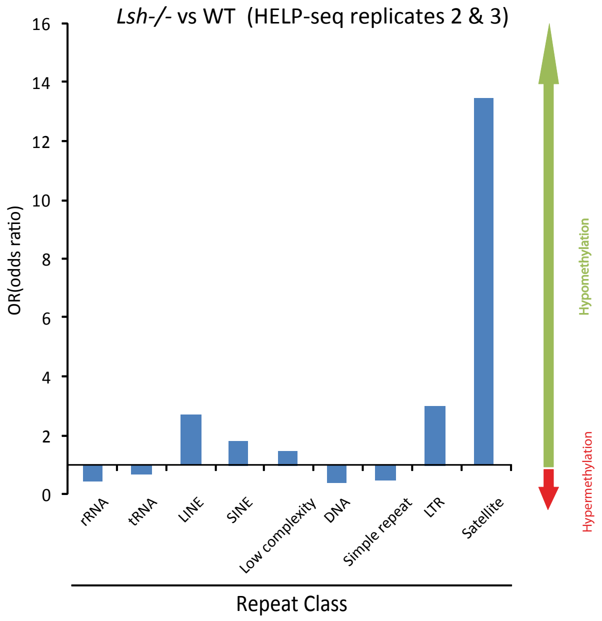


f)

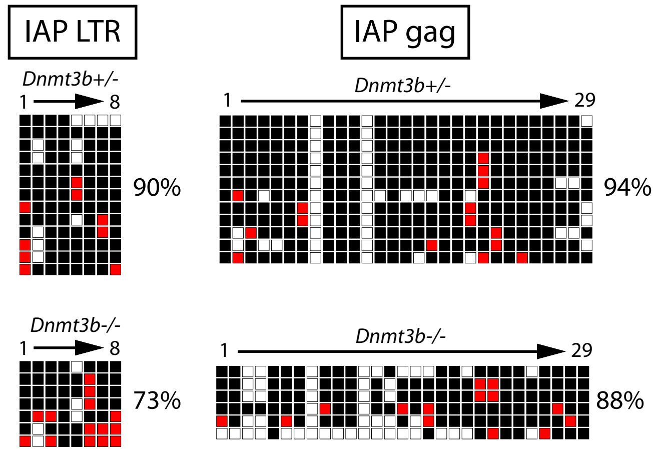


e)

| Reps 2 & 3 | | | | | | | | | |
|---|---|---|---|---|---|---|---|---|---|
| Class | rRNA | tRNA | LINE | SINE | DNA | Low complexity | Simple repeat | LTR | Satellite |
| *Lsh -/-* | 88030 | 4285 | 3213238 | 903925 | 74772 | 275181 | 160680 | 1646044 | 148105 |
| *Lsh -/-* proportion | 0.006078 | 0.000296 | 0.221863 | 0.062413 | 0.005163 | 0.019000335 | 0.011094421 | 0.113654 | 0.010226 |
| WT | 141381 | 4674 | 885197 | 365806 | 38262 | 531685 | 250854 | 408561 | 8211 |
| WT proportion | 0.013112 | 0.000433 | 0.082095 | 0.033926 | 0.003548 | 0.049309591 | 0.02326473 | 0.037891 | 0.000762 |
| OR | 0.463559 | 0.68254 | 2.702519 | 1.839702 | 1.454913 | 0.385327377 | 0.47687727 | 2.99951 | 13.42887 |

g)

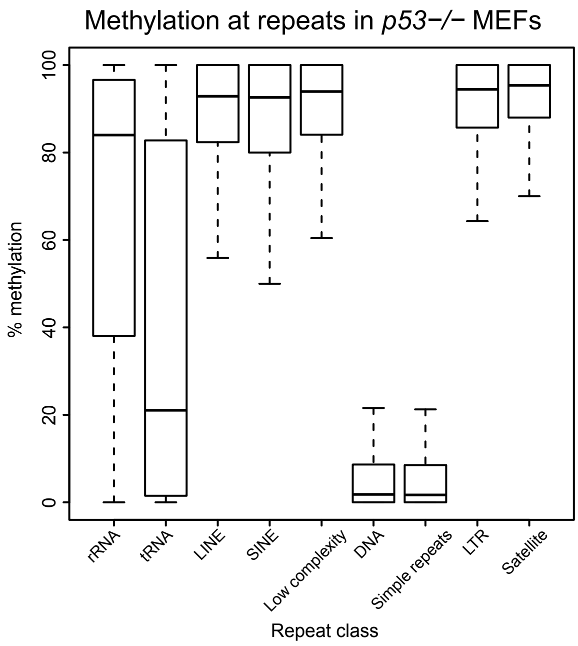


h)

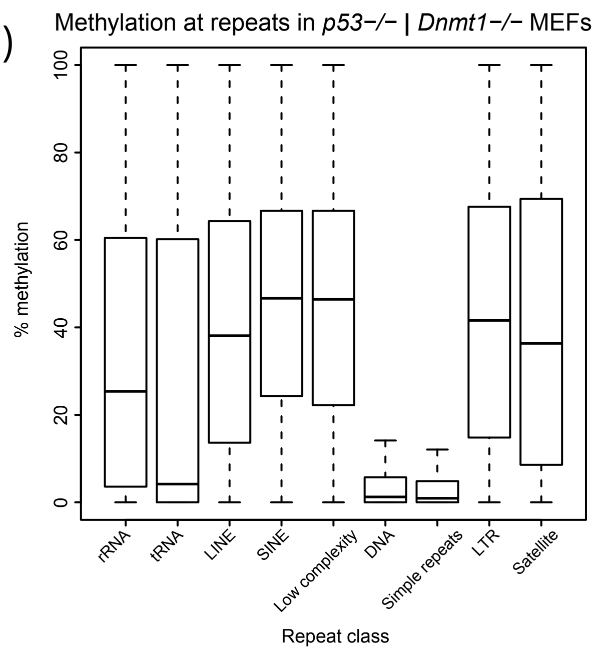

# Supp S3

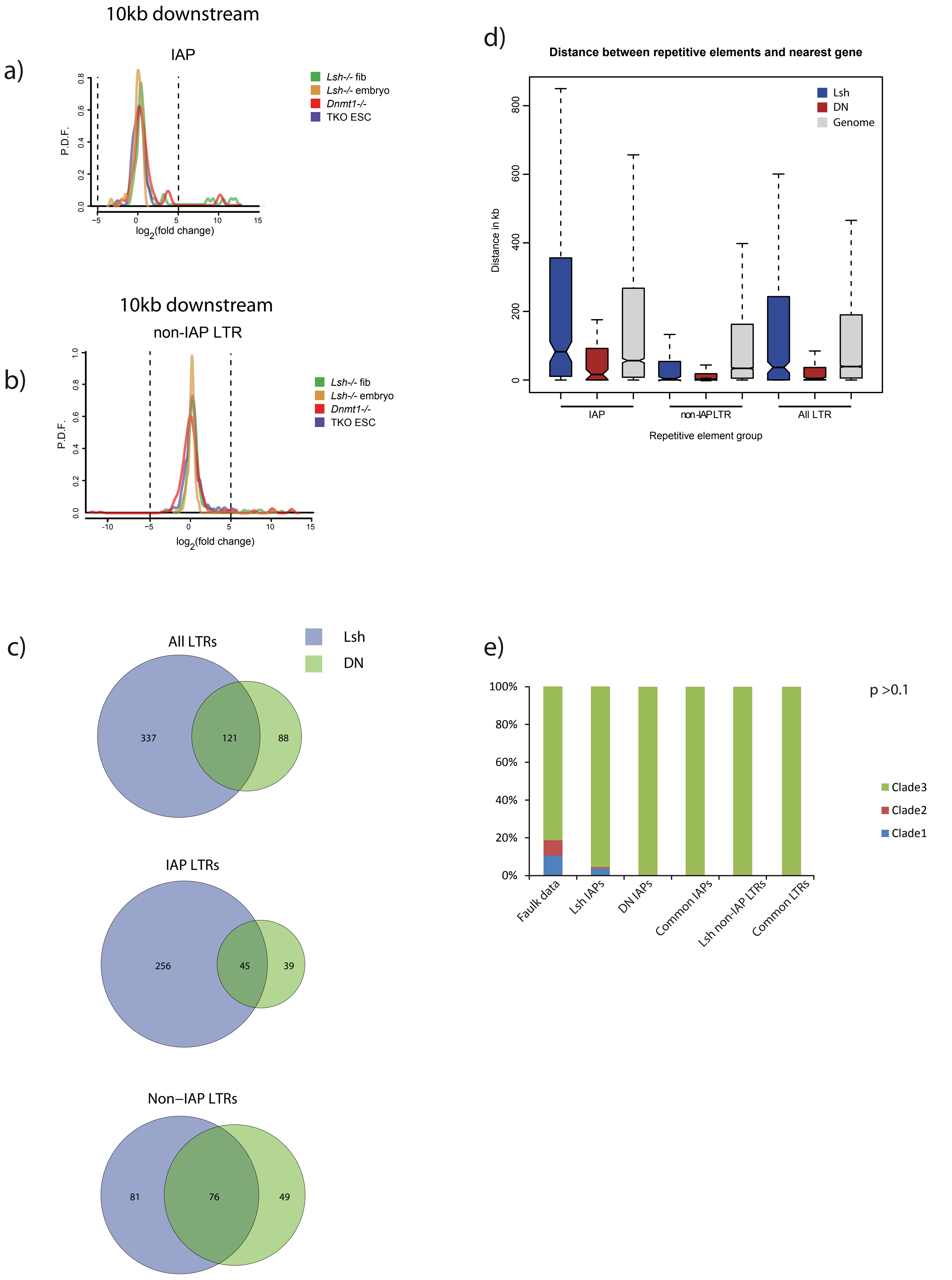

10kb downstream
a)
IAP
P.D.F.
0.0
0.2
0.4
0.6
0.8
-5
0
5
10
15
log2(fold change)
Lsh-/- fib
Lsh-/- embryo
Dnmt1-/-
TKO ESC
10kb downstream
non-IAP LTR
b)
P.D.F.
0.0
0.2
0.4
0.6
0.8
1.0
-10
-5
0
5
10
15
log2(fold change)
Lsh-/- fib
Lsh-/- embryo
Dnmt1-/-
TKO ESC
d)
Distance between repetitive elements and nearest gene
Distance in kb
0
200
400
600
800
Lsh
DN
Genome
IAP
non-IAP LTR
All LTR
Repetitive element group
c)
All LTRs
Lsh
DN
337
121
88
IAP LTRs
256
45
39
Non–IAP LTRs
81
76
49
e)
100%
80%
60%
40%
20%
0%
p >0.1
Clade3
Clade2
Clade1
Faulk data
Lsh IAPs
DN IAPs
Common IAPs
Lsh non-IAP LTRs
Common LTRs

# Supp S4

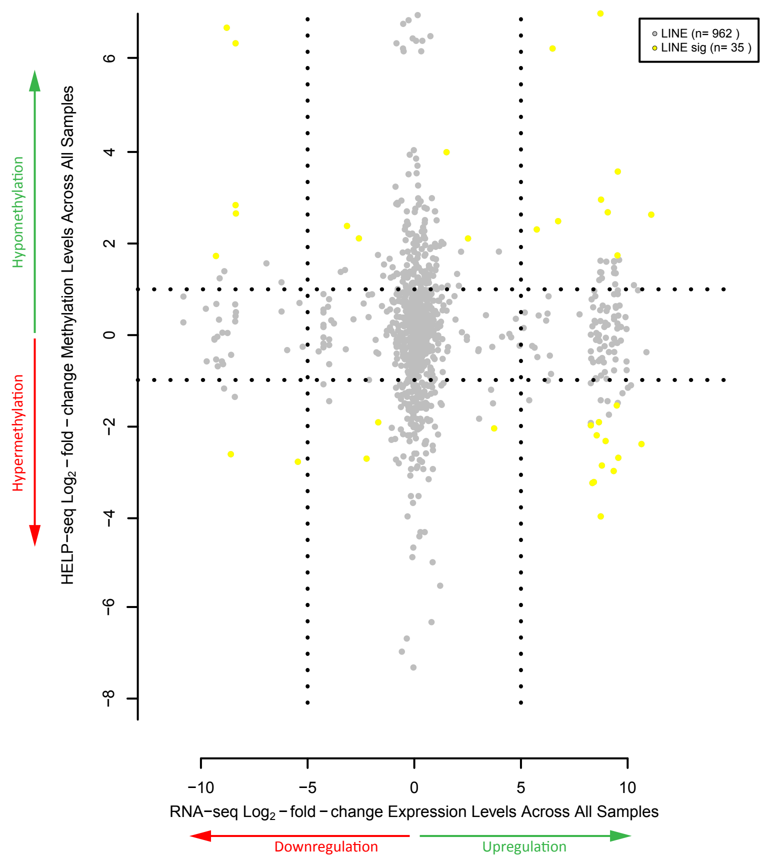

LINE (n= 962 )
LINE sig (n= 35 )
HELP−seq Log2 − fold − change Methylation Levels Across All Samples
Hypomethylation
Hypermethylation
RNA−seq Log2 − fold − change Expression Levels Across All Samples
Downregulation
Upregulation

# Supp S5

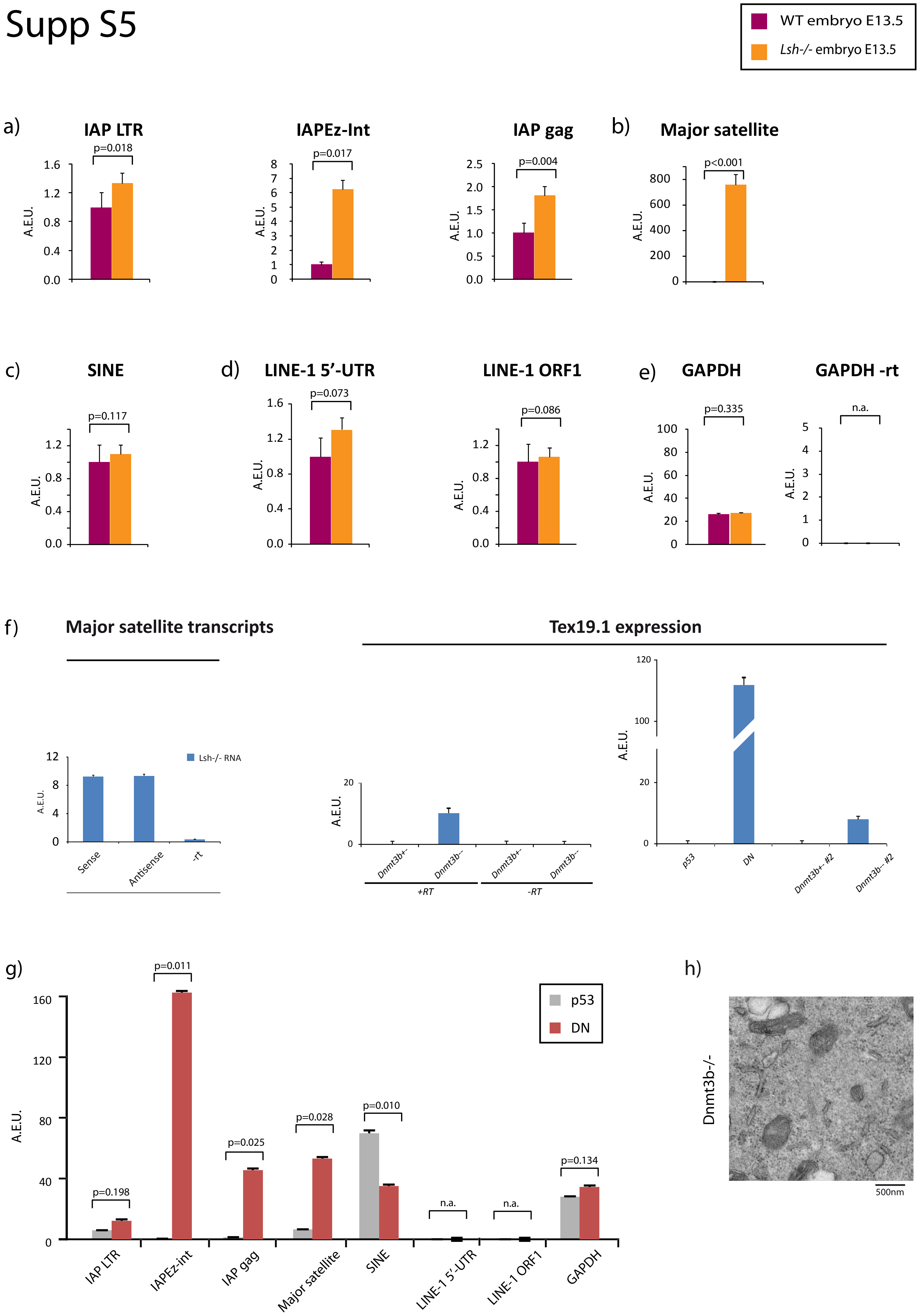

WT embryo E13.5
Lsh-/- embryo E13.5
a)
IAP LTR
p=0.018
A.E.U.
1.6
1.2
0.8
0.4
0.0
IAPEz-Int
p=0.017
8
7
6
5
4
3
2
1
0
IAP gag
p=0.004
2.5
2.0
1.5
1.0
0.5
0.0
b)
Major satellite
p<0.001
800
600
400
200
0
c)
SINE
p=0.117
d)
LINE-1 5'-UTR
p=0.073
LINE-1 ORF1
p=0.086
e)
GAPDH
p=0.335
100
80
60
40
20
GAPDH -rt
n.a.
5
4
3
2
1
f)
Major satellite transcripts
Lsh-/- RNA
12
8
4
Sense
Antisense
-rt
Tex19.1 expression
20
Dnmt3b+/-
Dnmt3b-/-
+RT
-RT
120
100
p53
DN
Dnmt3b+/- #2
Dnmt3b-/- #2
g)
p53
DN
p=0.011
p=0.198
p=0.025
p=0.028
p=0.010
n.a.
p=0.134
160
120
80
40
IAP LTR
IAPEz-int
IAP gag
Major satellite
SINE
LINE-1 5'-UTR
LINE-1 ORF1
GAPDH
h)
Dnmt3b-/-
500nm

# Supp S6

a)

## Bisulfite primer regions

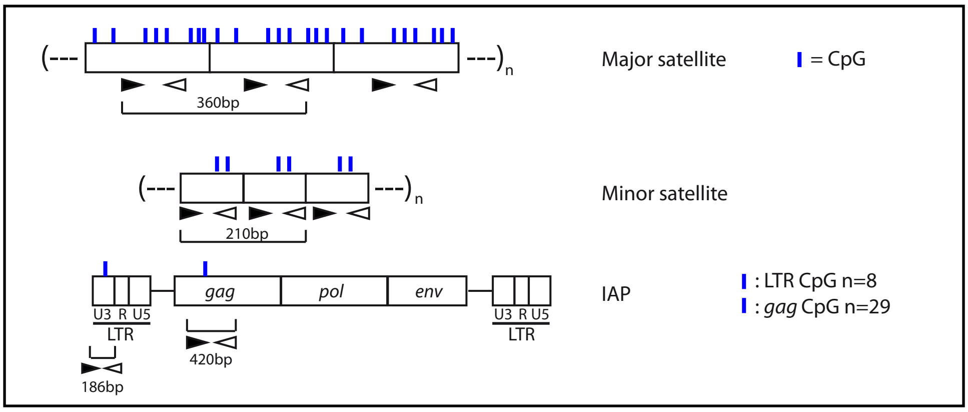


b)

## qRTPCR primer regions

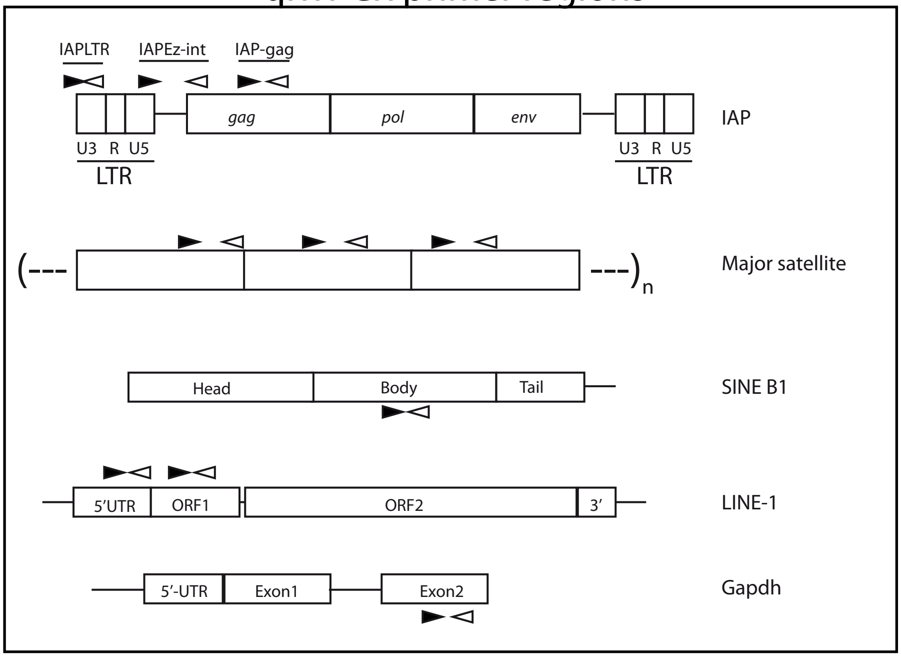


c)

## ChIP primer regions

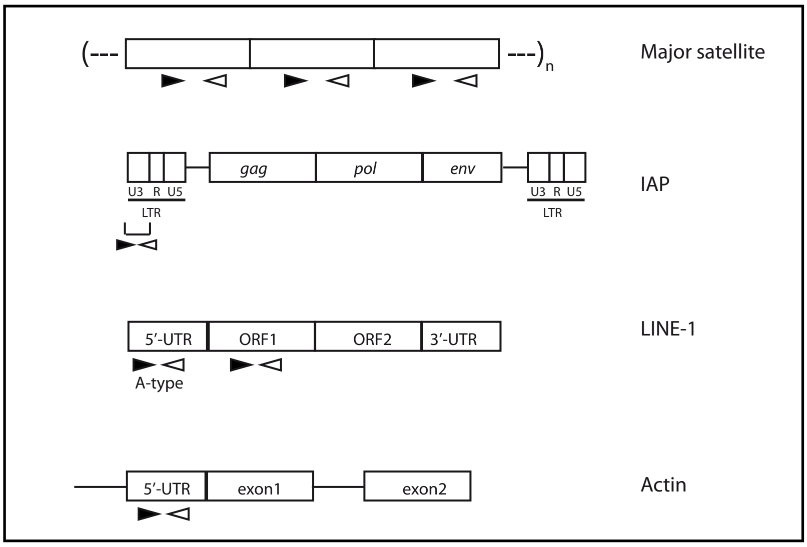

# Supp S7

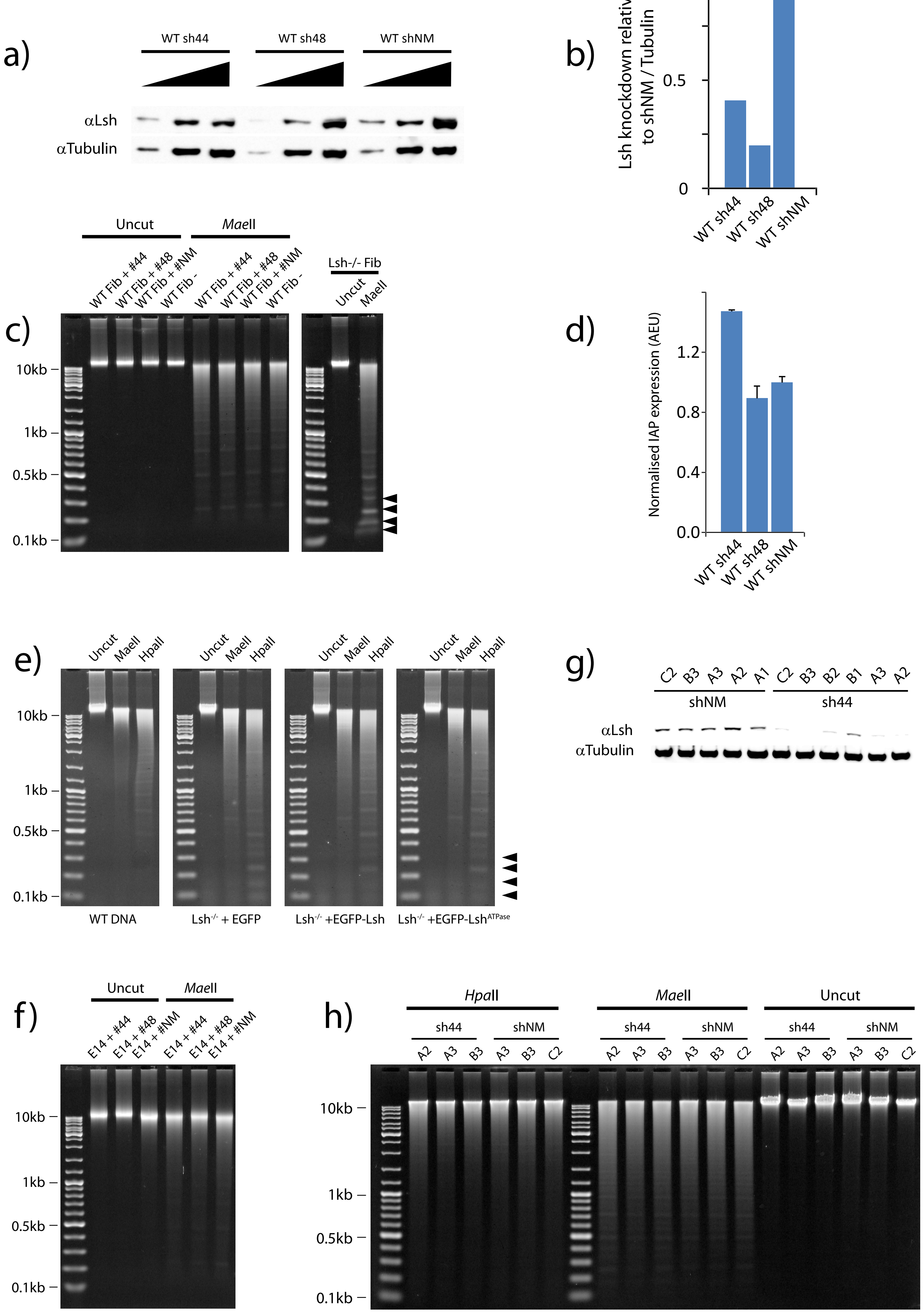

a)
WT sh44
WT sh48
WT shNM
αLsh
αTubulin
b)
Lsh knockdown relative to shNM / Tubulin
1
0.5
0
WT sh44
WT sh48
WT shNM
c)
Uncut
MaeII
WT Fib + #44
WT Fib + #48
WT Fib + #NM
WT Fib -
Lsh-/- Fib
Uncut
MaeII
10kb
1kb
0.5kb
0.1kb
d)
Normalised IAP expression (AEU)
1.2
0.8
0.4
0.0
WT sh44
WT sh48
WT shNM
e)
Uncut
MaeII
HpaII
10kb
1kb
0.5kb
0.1kb
WT DNA
Lsh-/- + EGFP
Lsh-/- +EGFP-Lsh
Lsh-/- +EGFP-LshATPase
g)
C2
B3
A3
A2
A1
C2
B3
B2
B1
A3
A2
shNM
sh44
αLsh
αTubulin
f)
Uncut
MaeII
E14 + #44
E14 + #48
E14 + #NM
10kb
1kb
0.5kb
0.1kb
h)
HpaII
MaeII
Uncut
sh44
shNM
A2
A3
B3
A3
B3
C2
10kb
1kb
0.5kb
0.1kb

Supp. S1 List of primer oligonucleotides used in this study

Bisulfite sequencing

| Name | Forward | Reverse |
|---|---|---|
| CHR2:95100568-95100802 outer | TGTGTTTTTGTTTGTTTTAG | AAACAACACCCTTTATAAAC |
| CHR2:95100568-95100802 inner | TTGTTTTAGAAGTTGTTAGG | AACCTAACAACTTCTAAAAC |
| CHR15:31097720-3109793 outer | ATGAATTTAATTAGTGGTTG | CTACTACAAACCAAAAAACC |
| CHR15:31097720-3109793 inner | GTGGTTGTGGTGTTTTTAAG | TCAAAAAACTACTACAAACC |
| Major satellite | GGAATATGGTAAGAAAATTGAAAATTATGG | CCATATTCCAAATCCTTCAATATACATTTC |
| Minor satellite | TAGAATATATTAGATGAGTGAGTTATATTG | ATTATAACTCATTAATATACACTATTCTAC |
| IAP_gag outer | TTTTGAGGAAATAGATTGGGAG | TCAACAAACRTTAATACCATAC |
| IAP_gag inner | AGATTGGGAGGAAGAAGTAG | CAACRAACCTATCTAACTAC |
| IAP_ltr outer | GGGTGGTTTATTTGTTTTTATTAAAAG | AAACRCATCACTCCCTAATTAACTAC |
| IAP_ltr inner | GTTTTTATTAAAAGAAAAAGGGG | ATTAACTACAACCCATAACC |

qRTPCR

| Name | Forward | Reverse |
|---|---|---|
| IAP LTR | GATGGTGCTGACATCCTGTG | CTGACGTTCACGGGAAAAAC |
| IAP-EzInt | TCCCCAGCTGAAAAGTTCTG | AAGAGAAAATCCGGGACGAG |
| IAP gag | TCCACGCTCCGGTAGAATAC | AGGCGTTAGTGCCATACCAG |
| Major satellite | AAATACACACTTTAGGACG | TCAAGTGGATGTTTCTCATT |
| Sine | CGCCTTTAATTGCAGCACTC | TGTAGAGCCCTAGCTGTCCTG |
| L1-5'UTR | AATCTGTCTCCCAGGTCTGC | CCTTTCGCCATCTGGTAATC |
| L1 ORF1 | ATCCAGGAAATCCAGGACAC | TTTGCTGGACCTTTGAGTTG |
| GAPDH | GGTCCTCAGTGTAGCCCAAG | ACCCAGAAGACTGTGGATGG |

Strand-specific qRTPCR

| | | |
|---|---|---|
| Major satellite | AAATACACACTTTAGGACG | TCAAGTGGATGTTTCTCATT |
